# Effect of the naphthylene linker on the *J*‐aggregation abilities of chlorophyll‐*a* derivatives

**DOI:** 10.1111/php.14076

**Published:** 2025-02-13

**Authors:** Yuma Hisahara, Takeo Nakano, Hitoshi Tamiaki

**Affiliations:** ^1^ Graduate School of Life Sciences Ritsumeikan University Kusatsu Shiga Japan; ^2^ Department of Chemistry, Faculty of Science Shinshu University Nagano Japan

**Keywords:** chlorophyll, chlorosome, electronic absorption, *J*‐aggregate, naphthalene spacer

## Abstract

Chlorophyll(Chl)‐*a* derivatives inserting an ethynylene‐naphthylene linker between the chlorin π‐skeleton and hydroxymethyl group were prepared as models of chlorosomal Chls. Their syntheses were achieved via Sonogashira coupling reaction. Their *J*‐aggregation behaviors were investigated by electronic absorption and circular dichroism spectroscopic measurements. These studies revealed that the 2,6‐naphthylene inserted Chl‐*a* derivatives gave the single *J*‐aggregation species in an aqueous Triton X‐100 micellar solution with a larger red‐shift value (1270 cm^−1^) of the Qy band in spite of its longer linker compared with *p*‐phenylene inserted Chl‐*a* derivative (970 cm^−1^). These unique optical properties were also discussed based on the computational studies, which indicated the different positional relation of chlorin rings in the assemblies by the linker structure.

## Graphical abstract

Chlorophyll(Chl)‐*a* derivatives inserting an ethynylene‐naphthylene linker between the chlorin π‐skeleton and hydroxymethyl group were prepared, and their *J*‐aggregation behaviors were investigated by electronic absorption and circular dichroism spectroscopic measurements. These studies revealed that the 2,6‐naphthylene inserted Chl‐*a* derivatives gave a single *J*‐aggregation species in an aqueous Triton X‐100 micellar solution with a larger red‐shift value of the Qy band despite its longer linker compared with the *p*‐phenylene inserted Chl‐*a* derivative reported previously. This result suggested that the linkers could control the positional relationship of the chlorin rings in the self‐aggregates, which have a huge effect on the exciton splitting energies.

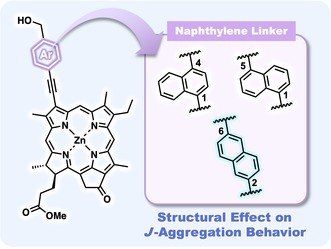

**Abbreviations**
BChl: bacteriochlorophyll
CD: circular dichroism
Chl: chlorophyll
DLS: dynamic light scattering
THF: tetrahydrofuran
TX‐100: Triton X‐100

## INTRODUCTION

Supramolecules are generally formed by electrostatic interaction, dipolar interaction, hydrogen bonding, and hydrophobic interaction. Their shapes depend in a large part on the structures of their building blocks, and they exhibit drastically different physical, electronical, and optical properties compared to those of monomers. Therefore, various investigation of supramolecules toward application in material chemistry, such as host‐guest systems,^1^, ^2^ self‐healing materials,^3^, ^4^, ^5^ redox catalysts,^6^ nano‐machines,^7^, ^8^ and drug‐delivery systems.^9^, ^10^ The supramolecular features are also important in the natural world. For instance, *J*‐aggregates of bacteriochlorophyll (BChl) molecules act as light‐harvesting antenna, which are essential for photosynthesis.^11^, ^12^, ^13^, ^14^, ^15^, ^16^, ^17^, ^18^, ^19^, ^20^, ^21^, ^22^, ^23^, ^24^, ^25^, ^26^, ^27^, ^28^, ^29^, ^30^, ^31^, ^32^, ^33^, ^34^, ^35^, ^36^, ^37^ This antenna consisting of BChl‐*c*, BChl‐*d*, BChl‐*e*, and BChl‐*f* is called as “chlorosome”,^38^, ^39^, ^40^, ^41^, ^42^, ^43^ and the exploration of chlorosomal antenna based on the synthetic chlorophyll (Chl) molecules are required to realize artificial photosynthesis.

**FIGURE 1:**
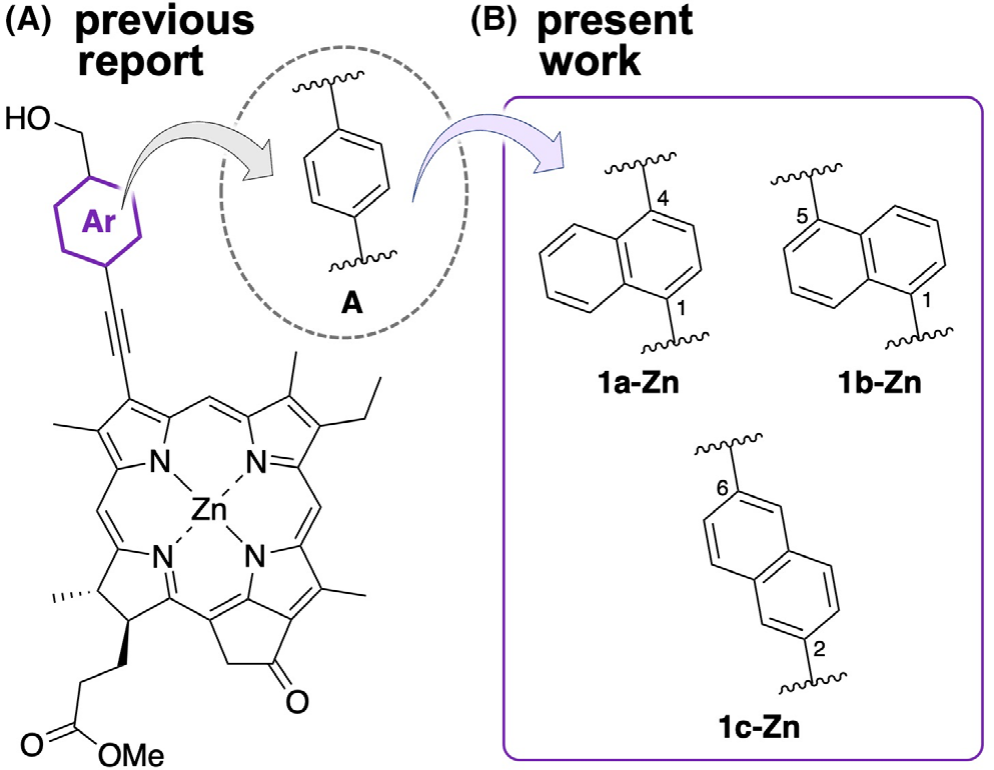
Ethynylene‐*p*‐phenylene linker inserted Chl‐*a* derivative **A** (A) and ethynylene‐naphthylene linker inserted Chl‐*a* derivatives **1a‐Zn**, **1b‐Zn**, and **1c‐Zn** in the present work.

Naturally occurring BChl molecules give their *J*‐aggregates through hydrogen bond (3^1^‐OH and 13‐C=O) and coordination bond (3^1^‐O and Mg).^44^, ^45^, ^46^ Therefore, synthesis of Chl molecules bearing the similar functional groups could afford the novel building blocks for the chlorosomal photosynthetic antennae. Actually, various Chl molecules bearing various functional groups at their peripheral positions, such as C3^1^‐, C7‐, C8‐, C13^2^‐, C17‐, and C20‐positions,^47^, ^48^, ^49^, ^50^, ^51^, ^52^, ^53^, ^54^, ^55^, ^56^, ^57^, ^58^, ^59^, ^60^, ^61^, ^62^ were synthesized based on zinc 3^1^‐hydroxy‐13^1^‐oxo‐chlorin^63^, ^64^, ^65^ to investigate their *J*‐aggregation abilities. Further, Chl‐*a* derivatives inserting an ethynylene and phenylene linker between the hydroxymethyl group and chlorin π‐skeleton were also prepared to control the exciton splitting energies based on a chlorin‐to‐chlorin distance in the resulting self‐aggregates (Figure 1A).^66^, ^67^, ^68^, ^69^ These studies revealed that the position of the hydroxymethyl group and substituents on the phenylene group were influence on their *J*‐aggregation behaviors. Thus, the structure of the linker could be fundamental for the control of *J*‐aggregation abilities of such Chl‐*a* derivatives and optical properties of the resulting self‐aggregates, which is essential for development of the artificial light‐harvesting antenna. Especially, the previous reports exhibited that the linear linkers, including an ethynylene‐*p*‐phenylene moiety, gave Chl‐*a* derivatives good *J*‐aggregation abilities.^66^ Based on these studies, we herein designed and synthesized the Chl‐*a* derivatives inserting a naphthylene linker as the π‐expanded skeleton with a linear‐like structure; we focused the 1,4‐, 1,5‐, and 2,6‐naphthylene linker in which the two substituted C‐C bonds on the naphthalene ring are in parallel (Figure 1B). Their *J*‐aggregation behaviors were investigated by electronic absorption and circular dichroism (CD) spectroscopy in an aqueous micellar solution.

## RESULTS AND DISCUSSION

### Synthesis of Chl‐*a* derivatives 1‐Zn

**SCHEME 1:**
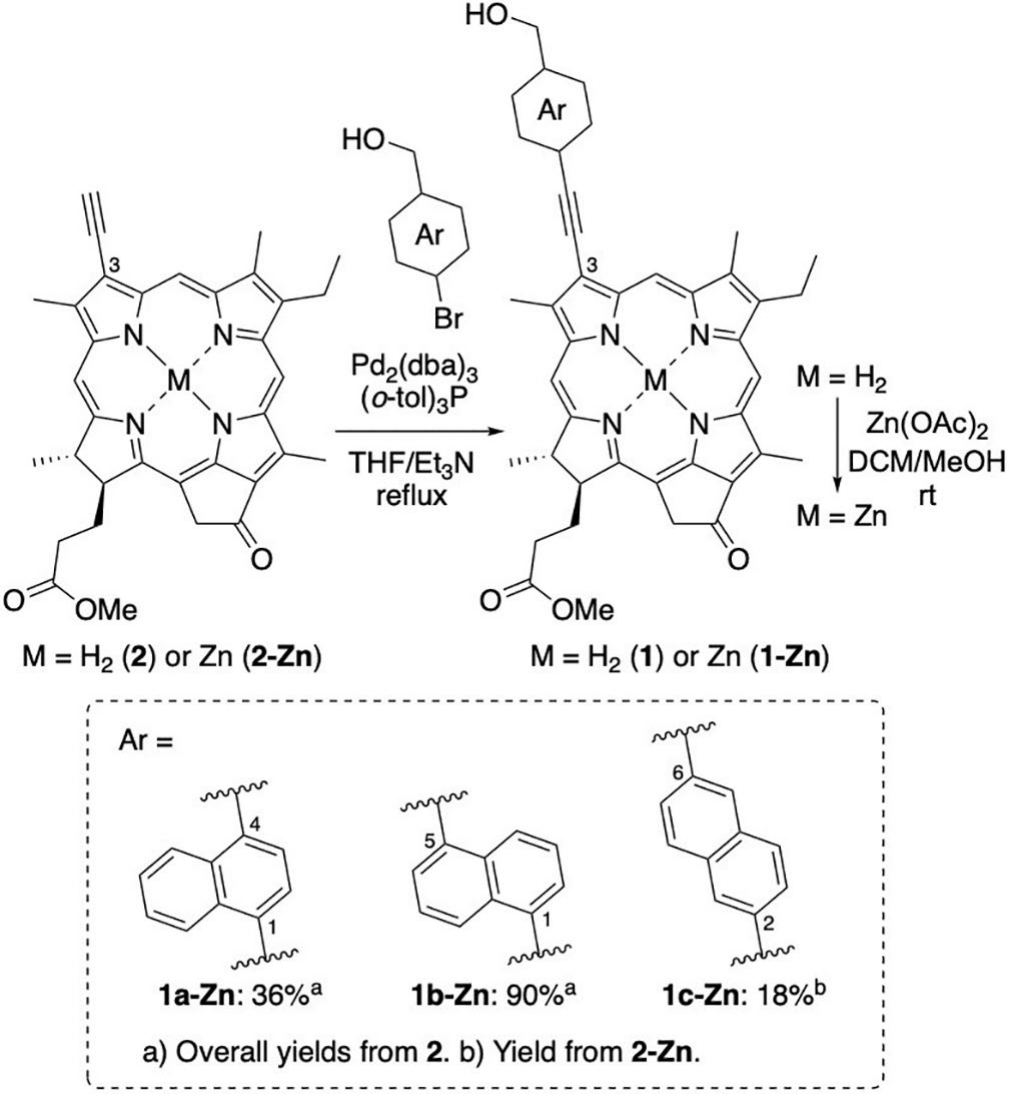
Synthesis of Chl‐*a* derivatives bearing a (hydroxymethylated naphthyl)ethynyl group at the C3 position: dba, dibenzylideneacetone; DCM, dichloromethane; THF, tetrahydrofuran; tol, tolyl.

Naphthylene and ethynylene linker inserted Chl‐*a* derivatives **1‐Zn** were prepared from C3‐ethynylated Chl‐*a* derivatives **2** or **2‐Zn** via Sonogashira coupling reaction as shown in Scheme 1.^70^, ^71^, ^72^ The free base of Chl‐*a* derivative **1a** inserting a 1,4‐naphthylene linker was synthesized via Pd‐catalyzed Sonogashira reaction of **2** with 1‐bromo‐4‐(hydroxymethyl)naphthalene. After that, regular zinc metalation^70^ furnished zinc complex **1a‐Zn**. In a similar way of **2** → **1a** → **1a‐Zn**, the Chl‐*a* derivative **1b‐Zn** inserting a 1,5‐naphthylene linker was synthesized from **2** and 1‐bromo‐5‐(hydroxymethyl)naphthalene. Although the Chl‐*a* derivative **1c** was formed via Sonogashira coupling of **2** with 2‐bromo‐6‐(hydroxymethyl)naphthalene, its isolation was difficult due to the inseparable impurities. Therefore, **1c‐Zn** was synthesized by the coupling of **2‐Zn** with 2‐bromo‐6‐(hydroxymethyl)naphthalene after the zinc metalation of **2**. The synthetic details with their spectral data were described in the Materials and method section of Supporting Information.

### Self‐aggregation of Chl‐*a* derivatives in an aqueous micellar solution

**FIGURE 2:**
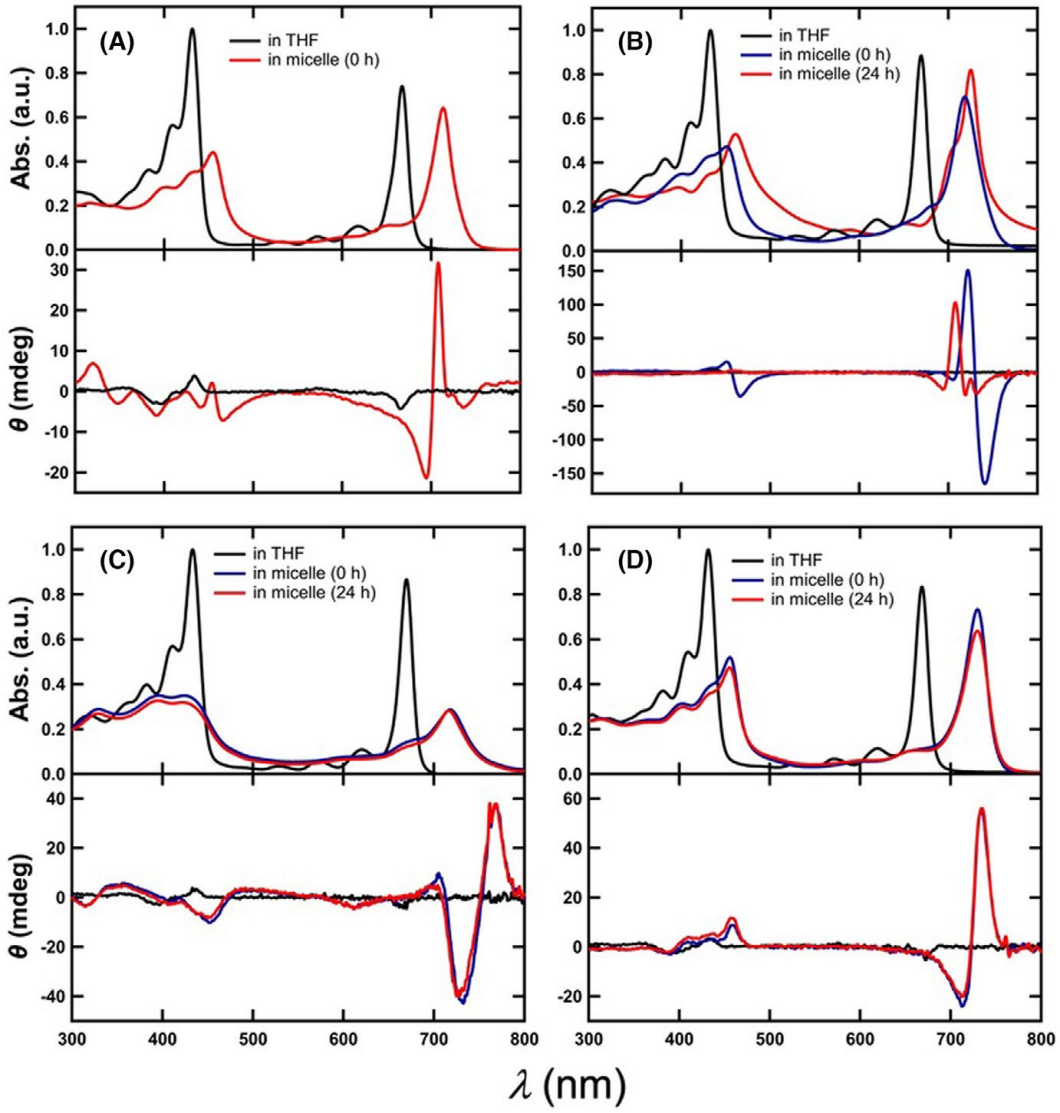
Electronic absorption (upper) and CD spectra (lower) of **A** (A), **1a‐Zn** (B), **1b‐Zn** (C), and **1c‐Zn** (D) in THF and an aqueous solution of 0.025% (wt/v) TX‐100 and 1% (v/v) THF.

In our previous report, the electronic absorption and CD spectra of *p*‐phenylene inserted Chl‐*a* derivative **A** were measured (Figure 2A).^66^ The electronic absorption spectrum of **A** in tetrahydrofuran (THF) exhibited large and sharp bands at 431 and 667 nm (Table 1), which are called Soret and Qy bands, respectively (Figure 2A, upper, black line). The spectrum was characterized as monomeric **A** with single axial THF coordinated to the central Zn atom. Meanwhile, a freshly prepared aqueous Triton X‐100 (TX‐100) micellar solution of **A** exhibited red‐shifted Soret and Qy bands relative to those in THF; *λ* ^Soret^/*λ* ^Qy^ = 455/713 nm (Figure 2A, upper, red line). Strong CD signals were observed at the red‐shifted Qy region (Figure 2A, lower, red line). These results indicated chlorosomal *J*‐aggregation of **A** in the aqueous micellar solution, based on the previous data observed for its related compounds.^73^, ^74^, ^75^

**TABLE 1:** Electronic absorption properties of **A**, **1a‐Zn**, **1b‐Zn**, and **1c‐Zn**.

Chl	*λ*_max_(monomer)^a^/nm		*λ*_max_(aggregate)^b^/nm	
	Soret	Qy	Soret	Qy
**A**	431	667	455	713
**1a‐Zn**	433	669	461	725
**1b‐Zn**	434	670	424	718
**1c‐Zn**	432	668	456	730

^a^ In THF.

^b^ In an aqueous solution of 0.025% (wt/v) TX‐100 and 1% (v/v) THF.

In the same manner, the self‐aggregation behaviors of the naphthylene‐inserted Chl‐*a* derivatives **1‐Zn** were investigated (Figure 2B–D). Monomeric **1a‐Zn** in THF exhibited similar but slightly red‐shifted Soret (433 nm) and Qy bands (669 nm) (Figure 2B, upper, black line) in comparison with those of **A**. These shifts were ascribed to the expanded π‐conjugated system by inserting a 1,4‐naphthylene group at the C3^2^‐position. An aqueous micellar solution of **1a‐Zn** exhibited a red‐shifted Qy band at 719 nm just after preparation of the aqueous solution (Figure 2B, upper, blue line). This behavior was similar to that of **A** in an aqueous micellar solution; however, the Qy band of **1a‐Zn** was changed over time. Namely, the Qy band at 719 nm decreased, and concomitantly the broadened band at 725 nm accompanying a shoulder at 705 nm increased (Figure 2B, upper, red line). Although the reason for this change is unclear, the 1,4‐naphthylene group could affect the stability of the self‐assembly, gradually changing from kinetically controlled (719‐nm absorbing) to thermodynamically controlled (725‐nm absorbing) self‐aggregates. Actually, **1a‐Zn** in an aqueous micellar solution gave the similar electronic absorption spectrum after standing for 90 min at 55°C.* However, the broadened absorption bands could indicate the disordered self‐assemblies including various species. The presence of strong bisignate CD signals in the Qy region just after preparation of the aqueous micellar solution was indicative of the *J*‐aggregation of **1a‐Zn** (Figure 2B, lower, blue line). On the other hand, the CD signals in the Qy region after standing for 24 h drastically decreased (Figure 2B, lower, red line); a positive signal was observed around 705 nm, in which the shoulder band appeared in the absorption spectrum. These changes of the CD signals were consistent with the formation of the different self‐assemblies, which were exhibited by the absorption spectra.

The 1,5‐naphthylene inserted Chl‐*a* derivative **1b‐Zn** also did not exhibit the well‐ordered *J*‐aggregation. Comparing its electronic absorption spectrum in THF (Figure 2C, upper, black line) with that in aqueous micellar solution (Figure 2C, upper, red line), the bathochromic shift was observed; *λ* ^Qy^ = 670 (in THF) < 718 nm (in micelle). The absorption bands in an aqueous micellar solution were significantly broadened, where red‐shifted bands were overlapped with the corresponding monomeric bands. These results suggested that some disordered self‐assemblies were generated,^76^ and the monomeric **1b‐Zn** had remained. The aggregated **1b‐Zn** showed the S‐shaped CD bands at the shifted regions (Figure 2C, lower, red line).

The *J*‐aggregation behavior of Chl‐*a* derivative **1c‐Zn** was also investigated (Figure 2D). In THF, monomeric **1c‐Zn** exhibited slightly red‐shifted Soret and Qy bands relative to those of **A** (Figure 2D, upper, black line); *λ* ^Soret^ = 432 (**1c‐Zn**) > 431 nm (**A**) and *λ* ^Qy^ = 668 (**1c‐Zn**) > 667 nm (**A**). These bathochromic shifts were due to the π‐expanded structure mentioned above. When **1c‐Zn** was dissolved in an aqueous TX‐100 micellar solution, largely red‐shifted Soret and Qy bands appeared in comparison with those in THF. In the Qy region, a remarkably shifted band was observed at 730 nm, and the spectrum hardly changed during standing for 24 h except the slight suppression of the 730‐nm intensity (Figure 2D, upper, blue to red line). This Qy band in an aqueous micellar solution was sharper than those of **1a/b‐Zn** and its full width at half maximum (564 cm^−1^) was comparable to that of **A** (550 cm^−1^), which suggested that single self‐assembly species of **1c‐Zn** was favorable in the aqueous micelle. A strong CD couplet at the red‐shifted Qy region indicated that the chlorosomal *J*‐aggregation of **1c‐Zn** occurred (Figure 2D, lower, red line), similarly as in **A**.

### Calculation studies of the exciton splitting energies in self‐aggregates of Chl‐*a* derivatives

**FIGURE 3:**
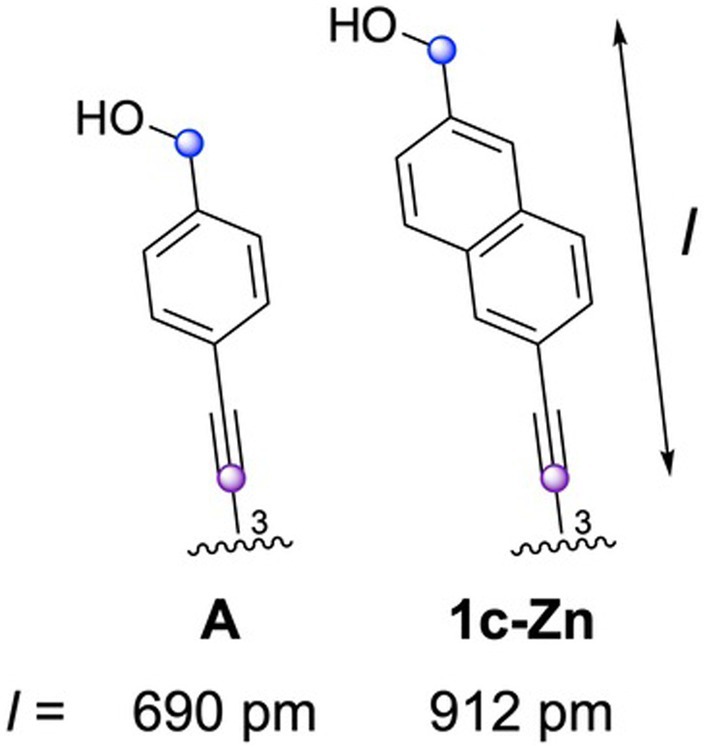
Estimated lengths (*l*) of linkers by MM+/PM3 calculations between the C3^1^ and hydroxymethyl carbon atoms of **A** and **1c‐Zn**.

**FIGURE 4:**
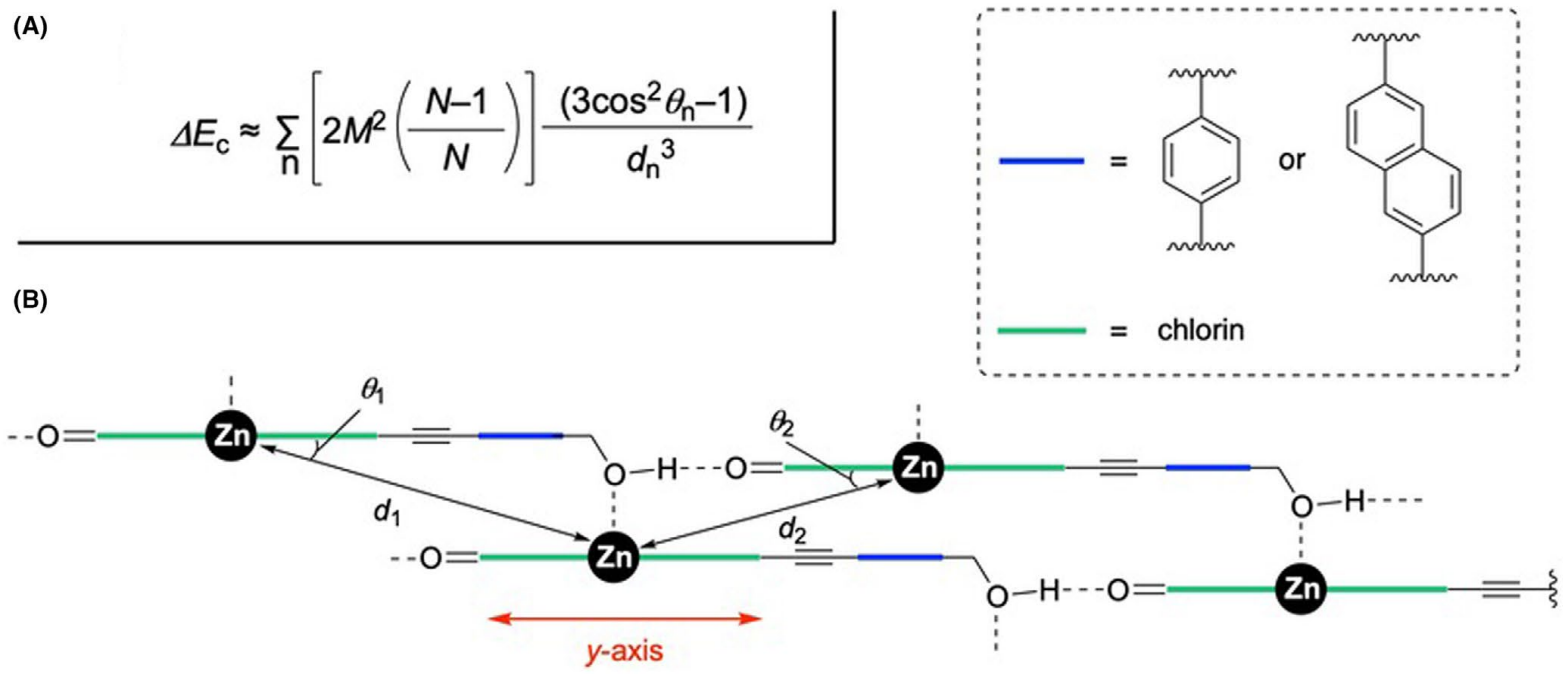
Distances (*d*) and angles (*θ*) between chlorin rings in *J*‐aggregates of **A** and **1c‐Zn**.

As shown in Figure 2, **1A‐Zn** and **1b‐Zn** gave the relatively disordered chlorosomal self‐aggregates in an aqueous micellar solution in comparison with **A** and **1c‐Zn**. These results could indicate that a hydroxymethyl group at the α‐position of a naphthalene ring linker put at a disadvantage to form the self‐aggregates. Therefore, exciton splitting energies (Δ*E*) of **A** and **1c‐Zn**, which gave their well‐ordered chlorosomal self‐aggregates in an aqueous micellar solution, were estimated by their red‐shift values (Δ*λ*) as shown in Table 2.† In our previous report,^66^ Δ*E* value of **A** was decreased in comparison with 3‐hydroxymethylated Chl‐*a* derivative due to the extended chlorin‐to‐chlorin distance in the expected aggregates by the inserted ethynylene‐*p*‐phenylene linker. However, Δ*E* value of **1c‐Zn** inserting a longer linker was larger than that of **A**; Δ*E* = 2.53 × 10^−20^ (**1c‐Zn**) > 1.92 × 10^−20^ J (**A**), namely, the ratio of Δ*E*(**1c‐Zn**) over Δ*E*(**A**) was 1.32. Actually, the calculation exhibited that the linker length of **1c‐Zn** was longer than that of **A** as shown in Figure 3; *l* = 912 (**1c‐Zn**) > 690 pm (**A**). These results suggested that the linker length is not necessarily a dominant factor to determine the Δ*E* value in the aggregates. For the further investigation, the calculation studies were carried out based on the expected chlorosomal self‐aggregate model of **A** and **1c‐Zn** (Figure 4). The relationship of the exciton splitting energy with the chlorin‐to‐chlorin distance *d* and angle *θ* was expressed as shown in Figure 4A; *M* represents the magnitude of the transition moment of the chlorin molecules, and *N* is the molecular number in their self‐aggregates.^77^ Regarding **A** and **1c‐Zn**, their main chlorin π‐skeletons were identical and their structural difference is the inserted phenylene/naphthylene linker between the hydroxymethyl and C3‐ethynyl group. Therefore, the values of *M* along the y‐axis (C3–C13 line) of **A** and **1c‐Zn** monomers were assumed to be comparable. The chlorin numbers *N* in **A** and **1c‐Zn** self‐aggregates were investigated based on the dynamic light scattering (DLS) studies.‡ These results exhibited that the self‐aggregate of **1c‐Zn** was larger than that of **A**, namely the chlorin number *N* of **1c‐Zn** aggregate was also larger that of **A** aggregate. However, these differences could have little effect on the value of the *N* − 1/*N* (≈1) due to the larger values for *N*s (*N* >> 1), which involve in the equation as shown in Figure 4A. These assumptions indicate that the calculated Δ*E* is dependent in (3cos^2^ *θ* − 1)/*d* ^3^ = *K*.

**TABLE 2:** Exciton splitting energies Δ*E* and red‐shift values Δ*λ* by self‐aggregation of zinc Chl‐*a* derivatives **A** and **1c‐Zn**.

Chl	*λ*_max_^Qy^/nm		Δ*λ*/cm^−1^	Δ*E*/10^−20^ J
	Monomer^a^	Aggregate^b^		
**A**	667	713	970	1.92
**1c‐Zn**	668	730	1270	2.53

^a^ In THF.

^b^ In an aqueous solution of 0.025% (wt/v) TX‐100 and 1% (v/v) THF.

The chlorosomal model derived from linker‐inserted Chl‐*a* derivative **A** and **1c‐Zn** were illustrated in Figure 4B, and their *K*‐values were determined as the sum of *K*_1_ and *K*_2_, which were estimated from *d*_1_ and *θ*_1_, and *d*_2_ and *θ*_2_, respectively. Our previous report exhibited that *K* for **A** was 1.91 × 10^27^ m^−3^.^66^ In a similar manner, *K* for **1c‐Zn** was determined by *d* and *θ*, which were estimated from the energy‐minimalized aggregate model by MM+ calculation; *d*_1_ = 1377 pm, *θ*_1_ = 25.6°, *d*_2_ = 881 pm, and *θ*_2_ = 31.5°. Based on these data, *K*_1_ and *K*_2_ values were calculated to be 5.51 × 10^26^ and 1.73 × 10^27^ m^−3^, respectively. Therefore, *K* for **1c‐Zn** was 2.23 × 10^27^ m^−3^. The ratio of *K*(**1c‐Zn**) over *K*(**A**) was 1.17, which was a little different from the aforementioned ratio Δ*E*(**1c‐Zn**)/Δ*E*(**A**) = 1.32, however, the order was identical. These results suggested that the model calculations were useful. Regarding the chlorin‐to‐chlorin distance, *d*(**1c‐Zn**) was larger than *d*(**A**) due to the longer naphthylene linker; *d*_1_ = 1377 (**1c‐Zn**) > 1231 (**A**) pm and *d*_2_ = 881 (**1c‐Zn**) > 838 (**A**) pm.§ These larger *d* could give a smaller *K* in **1c‐Zn**. By contrast, *θ*(**1c‐Zn**) was sufficiently smaller than *θ*(**A**); *θ*_1_ = 25.6° (**1c‐Zn**) < 33.1° (**A**) and *θ*_2_ = 31.5° (**1c‐Zn**) < 39.7° (**A**). These smaller angles produced larger cos*θ*‐values, which resulted in larger *K*. These two conflicting factors led to Δ*E*(**1c‐Zn**)/Δ*E*(**A**) > 1, suggesting that the linker structure could drastically affect the chlorin‐to‐chlorin angles, which is one of the dominant parameters to control the excitonic interaction.

## CONCLUSIONS

We synthesized Chl‐*a* derivatives **1a‐Zn**, **1b‐Zn**, and **1c‐Zn** inserting an ethynylene‐naphthylene linker between the hydroxymethyl group and chlorin ring. These syntheses have been achieved via Pd‐catalyzed cross‐coupling reaction of a C3‐ethynylated Chl‐*a* derivative with the corresponding naphthyl bromides. Their self‐aggregation behaviors were investigated by measurements of electronic absorption and CD spectra in aqueous TX‐100 micelles. Although **1a‐Zn** and **1b‐Zn** inserting 1,4‐ and 1,5‐naphthylene linkers, respectively, exhibited their reduced self‐aggregation abilities, **1c‐Zn** inserting a 2,6‐naphthylene linker gave the well‐ordered self‐aggregates. Interestingly, the larger red‐shift value of the aggregated Qy band in **1c‐Zn** in comparison with that in **A** inserting a smaller *p*‐phenylene group was observed in spite of the longer linker. Namely, the excitonic interaction between the chlorin π‐skeletons in the self‐aggregates of **1c‐Zn** was larger than that of **A**, while the chlorin‐to‐chlorin distance in the self‐aggregates was extended. The calculation studies based on the chlorosomal self‐aggregate model of **1c‐Zn** exhibited the smaller chlorin‐to‐chlorin angles in comparison with those of **A**, which could enhance the excitonic interaction. These results suggested that the linker structures could drastically change the positional relationship of the chlorin rings in the self‐aggregates, which could be useful to control the exciton splitting energies.

## AUTHOR CONTRIBUTIONS

**Yuma Hisahara**: Investigation. **Takeo Nakano**: Project administration, Writing – original draft. **Hitoshi Tamiaki**: Project administration, Supervision, Funding acquisition, Writing – review and editing.

## CONFLICT OF INTEREST STATEMENT

The authors declare no conflict of interest.

## Supporting information


Data S1.

## Data Availability

The data that supports the findings of this study are available in the supplementary material of this article.

## Footnotes

* The electronic absorption and CD spectra of **1a‐Zn** in an aqueous micellar solution were measured with increasing the temperature from 20 to 55°C over 150 min. These results are shown in Supporting Information.

^†^
Δ*E* (J) = *hc*[1/*λ*(in THF) − 1/*λ*(in micelle)] × 10^9^: *h* is Planck constant (6.63 × 10^−34^ Js), *c* is the velocity of light (3.00 × 10^8^ ms^−1^), and *λ* is the Qy maximum (nm).

^‡^
The results of DLS measurements of the *J*‐aggregates of **A** and **1c‐Zn** are shown in Supporting Information.

^§^
The Δ*d*_1_ (146 pm) was smaller than Δ*l* (222 pm) between **A** and **1c‐Zn**. This result implied that the actual *J*‐aggregates were curved, not straight. The model structure by the calculation studies were shown in Supporting Information.
