# Supplementary material for: Effect of the naphthylene linker on the J‐aggregation abilities of chlorophyll‐a derivatives
Source: Photochem Photobiol. 2025 Feb 13;101(6):1508–15. doi: 10.1111/php.14076 (PMC12621082; doi:10.1111/php.14076)
Supplement: Supplementary file 1 — Data S1. [file PHP-101-1508-s001.docx]

**Supporting Information**

**Effect of the Naphthylene Linker**

**on the *J*-aggregation Abilities of Chlorophyll-*a* Derivatives**

Yuma Hisahara^1^, Takeo Nakano*^1,2^, and Hitoshi Tamiaki*^1^

*^1^* *Graduate School of Life Sciences, Ritsumeikan University, Kusatsu, Shiga 525-8577, Japan*

*^2^ Department of Chemistry, Faculty of Science, Shinshu University, 3-1-1 Asahi, Matsumoto, Nagano 390-8621, Japan*

*Email: t_nakano@shinshu-u.ac.jp (T. Nakano), tamiaki@fc.ritsumei.ac.jp (H. Tamiaki)*

**List of contents**

| Materials and methods | S2–S5 |
| --- | --- |
| ^1^H NMR spectra of Chl-*a* derivatives **1a**(**-Zn**), **1b**(**-Zn**), and **1c-Zn** | S6–S8 |
| HRMS of Chl-*a* derivatives **1a**(**-Zn**), **1b**(**-Zn**), and **1c-Zn** | S9–S10 |
| Electronic absorption and CD spectra of **1a-Zn** at 55 °C | S11 |
| Dynamic light scattering (DLS) data of **A** and **1c-Zn** | S11 |
| Model calculation data of **1c-Zn** monomer and tetramer | S12–S29 |

**Materials and methods**

**General**

All the melting points were measured with a Yanaco MP-S3 micro melting point apparatus. ^1^H NMR spectra were recorded in CDCl_3_ using JEOL AL400 (400 MHz) and ECA-600 (600 MHz) spectrometers. Residual CHCl_3_ (δ = 7.26 ppm) was used as an internal standard. For the NMR measurements of zinc complexes, 1.5% (v/v) pyridine-*d*_5_ was added. All the ^1^H NMR peaks were assigned by two-dimensional techniques including correlated spectroscopy and nuclear Overhauser effect spectroscopy. Electronic absorption and CD spectra were measured by a Hitachi U-4100 spectrophotometer and a JASCO J-1500 spectrometer, respectively; in solution with a 10 mm quartz cell. High-resolution mass spectra (HRMS) were recorded on a Bruker micrOTOF II spectrometer; time of flight technique, atmospheric pressure chemical ionization (APCI) or electrospray ionization (ESI), and positive mode. DLS data were measured by Malvern Zetasizer Nano-ZS (ZEN3600).

**Synthesis of Chl-*a* derivatives**

*Methyl 3-([1-(4-hydroxymethyl)naphthyl]ethynyl)pyropheophorbide-a (****1a****)*: A solution of methyl 3-ethynylpyropheophorbide-*a*^s1^ (**2**, 78.4 mg, 143 μmol), 1-bromo-4-(hydroxymethyl)naphthalene (34.3 mg, 145 μmol), Pd_2_(dba)_3_ (32.2 mg, 35.2 μmol), and (*o*-tol)_3_P (45.7 mg, 150 μmol) in dry THF (12 mL) and Et_3_N (4.0 mL) was stirred at 65 °C in the dark under Ar for 13 h. After cooling down to room temperature, an aqueous (aq.) saturated (sat.) NaHCO_3_ solution was added into the reaction mixture, extracted with dichloromethane (DCM), and dried over Na_2_SO_4_. After evaporation, the residue was purified by silica gel column chromatography (Merck, Kieselgel 60, 0.040–0.063 mm, DCM/Et_2_O = 33/1 to 4/1 → DCM/MeOH = 200/1 to 67/1) and recrystallization (from DCM/hexane) to give **1a-H_2_** (61.7 mg, 87.8 μmol) in 61% yield as a black solid: mp 252–253 ℃; Vis (THF) λ_max_/nm = 681 (relative intensity, 0.71), 620 (0.07), 541 (0.12), 514 (0.13), 420 (1.00); ^1^H NMR (CDCl_3_, 600 MHz) δ/ppm = 9.22 (1H, s, 10-H), 8.30 (1H, s, 20-H), 8.24 (1H, d, *J* = 8 Hz, 5-H of 3^2^-1-naphthyl), 7.70 (1H, s, 5-H), 7.65 (1H, t, *J* = 8 Hz, 6-H of 3^2^-1-naphthyl), 7.56 (1H, d, *J* = 8 Hz, 8-H of 3^2^-1-naphthyl), 7.45 (1H, d, *J* = 7 Hz, 3-H of 3^2^-1-naphthyl), 7.36 (1H, t, *J* = 8 Hz, 7-H of 3^2^-1-naphthyl), 7.09 (1H, d, *J* = 7 Hz, 2-H of 3^2^-1-naphthyl), 5.19, 5.12 (each 1H, d, *J* = 13 Hz, 4-CH_2_ of 3^2^-1-naphthyl), 4.95, 4.75 (each 1H, d, *J* = 18 Hz, 13^1^-CH_2_), 4.53 (1H, br-q, *J* = 8 Hz, 18-H), 4.19 (1H, br-d, *J* = 10 Hz, 17-H), 3.76 (3H, s, 17^2^-CO_2_CH_3_), 3.63 (2H, q, *J* = 8 Hz, 8-CH_2_), 3.31 (3H, s, 12-CH_3_), 3.06 (1H, br-s, 4-COH of 3^2^-1-naphthyl), 2.91 (3H, s, 2-CH_3_), 2.55 (3H, s, 7-CH_3_), 2.83–2.77, 2.40–2.33 (each 1H, m, 17-CH_2_), 2.76–2.71, 2.57–2.52 (each 1H, m, 17^1^-CH_2_), 1.90 (3H, d, *J* = 8 Hz, 18-CH_3_), 1.70 (3H, t, *J* = 8 Hz, 8^1^-CH_3_), 0.33, −2.27 (each 1H, s, NH×2); HRMS (ESI) found: *m*/*z* = 703.3285, calcd for C_45_H_43_N_4_O_4_: MH^+^, 703.3279.

*Zinc methyl 3-([1-(4-hydroxymethyl)naphthyl]ethynyl)pyropheophorbide-a (****1a-Zn****)*: To a solution of **1a-H_2_** (19.2 mg, 27.3 μmol) in DCM (10 mL), sat. Zn(OAc)_2_·2H_2_O in MeOH (4.0 mL) was added at room temperature in the dark and stirred under Ar for 6 h. After addition of aq. sat. NaHCO_3_, the mixture was extracted with DCM and dried over Na_2_SO_4_. All the solvents were evaporated, and the residue was purified by recrystallization (from DCM/hexane) to give **1a-Zn** (12.7 mg, 16.6 μmol) in 60% yield as a dark green solid: mp > 300 ℃; Vis (THF) λ_max_/nm = 669 (relative intensity, 0.89), 620 (0.14), 572 (0.09), 528 (0.07), 433 (1.00); ^1^H NMR (1.5% pyridine-*d*_5_–CDCl_3_, 400 MHz) δ/ppm = 9.67 (1H, s, 5-H), 9.60 (1H, s, 10-H), 9.11 (1H, d, *J* = 8 Hz, 8-H of 3^2^-1-naphthyl), 8.42 (1H, s, 20-H), 8.27 (1H, d, *J* = 8 Hz, 3-H of 3^2^-1-naphthyl), 8.09 (1H, d, *J* = 8 Hz, 5-H of 3^2^-1-naphthyl), 7.82 (1H, br-t, *J* = 8 Hz, 7-H of 3^2^-1-naphthyl), 7.73 (1H, d, *J* = 8 Hz, 2-H of 3^2^-1-naphthyl), 7.71 (1H, br-t, *J* = 8 Hz, 6-H of 3^2^-1-naphthyl), 5.29 (2H, d, *J* = 4 Hz, 4-CH_2_ of 3^2^-1-naphthyl), 5.20, 5.07 (each 1H, d, *J* = 20 Hz, 13^1^-CH_2_), 4.43 (1H, dq, *J* = 2, 7 Hz, 18-H), 4.23 (1H, br-d, *J* = 8 Hz, 17-H), 3.77 (2H, q, *J* = 8 Hz, 8-CH_2_), 3.70 (3H, s, 12-CH_3_), 3.57 (3H, s, 2-CH_3_), 3.55 (3H, s, 17^2^-CO_2_CH_3_), 3.41 (1H, br-t, *J* = 4 Hz 4-COH of 3^2^-1-naphthyl), 3.33 (3H, s, 7-CH_3_), 2.62–2.54, 2.32–2.22 (each 1H, m, 17-CH_2_), 2.44–2.36, 2.03–1.95 (each 1H, m, 17^1^-CH_2_), 1.74 (3H, d, *J* = 7 Hz, 18-CH_3_), 1.72 (3H, t, *J* = 8 Hz, 8^1^-CH_3_); HRMS (ESI) found: *m*/*z* = 765.2401, calcd for C_45_H_41_N_4_O_4_Zn: MH^+^, 765.2414.

*Methyl 3-([1-(5-hydroxymethyl)naphthyl]ethynyl)pyropheophorbide-a (****1b****)*: A solution of **2** (57.6 mg, 105 μmol), 1-bromo-5-(hydroxymethyl)naphthalene (38.3 mg, 162 μmol), Pd_2_(dba)_3_ (22.7 mg, 24.8 μmol), and (*o*-tol)_3_P (42.8 mg, 141 μmol) in dry THF (13 mL) and Et_3_N (4.0 mL) was stirred at 65 °C in the dark under Ar for 1 h. After cooling down to room temperature, aq. sat. NaHCO_3_ solution was added into the reaction mixture, extracted with DCM, and dried over Na_2_SO_4_. After evaporation, the residue was purified by silica gel column chromatography (Merck, Kieselgel 60, 0.040–0.063 mm, DCM/MeOH = 200/1 to 100/1) and recrystallization (from DCM/hexane) to give **1b-H_2_** (72.4 mg, 103 μmol) in 98% yield as a black solid: mp 214–215 ℃; Vis (THF) λ_max_/nm = 681 (relative intensity, 0.69), 619 (0.08), 541 (0.12), 514 (0.13), 419 (1.00); ^1^H NMR (CDCl_3_, 600 MHz) δ/ppm = 9.46 (1H, s, 10-H), 8.76 (1H, s, 5-H), 8.53 (1H, s, 20-H), 8.32 (1H, d, *J* = 7 Hz, 6-H of 3^2^-1-naphthyl), 8.17 (1H, d, *J* = 8 Hz, 2-H of 3^2^-1-naphthyl), 7.80 (1H, d, *J* = 7 Hz, 8-H of 3^2^-1-naphthyl), 7.650 (1H, t, *J* = 7 Hz, 7-H of 3^2^-1-naphthyl), 7.649 (1H, d, *J* = 8 Hz, 4-H of 3^2^-1-naphthyl), 7.40 (1H, t, *J* = 8 Hz, 3-H of 3^2^-1-naphthyl), 5.27, 5.23 (each 1H, d, *J* = 13 Hz, 5-CH_2_ of 3^2^-1-naphthyl), 5.12, 4.90 (each 1H, d, *J* = 18 Hz, 13^1^-CH_2_), 4.54 (1H, br-q, *J* = 7 Hz, 18-H), 4.27 (1H, br-d, *J* = 9 Hz, 17-H), 3.67 (3H, s, 12-CH_3_), 3.63 (2H, q, *J* = 8 Hz, 8-CH_2_), 3.55 (3H, s, 17^2^-CO_2_CH_3_), 3.32 (3H, s, 2-CH_3_), 2.82 (3H, s, 7-CH_3_), 2.74–2.71, 2.39–2.35 (each 1H, m, 17-CH_2_), 2.65–2.60, 2.33–2.28 (each 1H, m, 17^1^-CH_2_), 2.45 (1H, br-s, 5-COH of 3^2^-1-naphthyl), 1.93 (3H, d, *J* = 7 Hz, 18-CH_3_), 1.70 (3H, t, *J* = 8 Hz, 8^1^-CH_3_), 0.37, −1.94 (each 1H, s, NH×2); HRMS (APCI) found: *m*/*z* = 703.3254, calcd for C_45_H_43_N_4_O_4_: MH^+^, 703.3279.

*Zinc methyl 3-([1-(5-hydroxymethyl)naphthyl]ethynyl)pyropheophorbide-a (****1b-Zn****)*: To a solution of **1b-H_2_** (53.4 mg, 76.0 μmol) in DCM (30 mL), sat. Zn(OAc)_2_·2H_2_O in MeOH (15 mL) was added at room temperature in the dark and stirred under Ar for 2 h. After addition of aq. sat. NaHCO_3_, the mixture was extracted with DCM and dried over Na_2_SO_4_. All the solvents were evaporated, and the residue was purified by recrystallization (from DCM/hexane) to give **1b-Zn** (54.1 mg, 70.6 μmol) in 92% yield as a dark green solid: mp > 300 ℃; Vis (THF) λ_max_/nm = 670 (relative intensity, 0.87), 621 (0.11), 572 (0.06), 530 (0.04), 434 (1.00); ^1^H NMR (1.5% pyridine-*d*_5_–CDCl_3_, 600 MHz) δ/ppm = 9.67 (1H, s, 5-H), 9.60 (1H, s, 10-H), 9.05 (1H, d, *J* = 8 Hz, 2-H of 3^2^-1-naphthyl), 8.42 (1H, s, 20-H), 8.28 (1H, d, *J* = 8 Hz, 6-H of 3^2^-1-naphthyl), 8.15 (1H, d, *J* = 8 Hz, 8-H of 3^2^-1-naphthyl), 7.76 (1H, t, *J* = 8 Hz, 3-H of 3^2^-1-naphthyl), 7.72 (1H, d, *J* = 8 Hz, 4-H of 3^2^-1-naphthyl), 7.69 (1H, t, *J* = 8 Hz, 7-H of 3^2^-1-naphthyl), 5.27 (2H, s, 5-CH_2_ of 3^2^-1-naphthyl), 5.20, 5.08 (each 1H, d, *J* = 19 Hz, 13^1^-CH_2_), 4.42 (1H, br-q, *J* = 8 Hz, 18-H), 4.22 (1H, br-d, *J* = 8 Hz, 17-H), 3.78 (2H, q, *J* = 8 Hz, 8-CH_2_), 3.70 (3H, s, 12-CH_3_), 3.57 (3H, s, 17^2^-CO_2_CH_3_), 3.55 (3H, s, 2-CH_3_), 3.33 (3H, s, 7-CH_3_), 2.59–2.55, 2.30–2.24 (each 1H, m, 17-CH_2_), 2.42–2.37, 2.01–1.96 (each 1H, m, 17^1^-CH_2_), 1.74 (3H, d, *J* = 8 Hz, 18-CH_3_), 1.72 (3H, *J* = 8 Hz, t, 8^1^-CH_3_) [The 5-COH signal of 3^2^-1-naphthyl was invisible.]; HRMS (ESI) found: *m*/*z* = 765.2415, calcd for C_45_H_41_N_4_O_4_Zn: MH^+^, 765.2414.

*Zinc methyl 3-([2-(6-hydroxymethyl)naphthyl]ethynyl)pyropheophorbide-a (****1c-Zn****)*: A solution of zinc methyl 3-ethynylpyropheophorbide-*a*^s1^ (**2-Zn**, 17.2 mg, 28.2 μmol), 2-bromo-6-(hydroxymethyl)naphthalene (35.7 mg, 151 μmol), Pd_2_(dba)_3_ (6.4 mg, 7.0 μmol), and (*o*-tol)_3_P (8.2 mg, 27.3 μmol) in dry THF (12 mL) and Et_3_N (4.0 mL) was stirred at 65 °C in the dark under Ar for 30 min. After cooling down to room temperature, aq. sat. NaHCO_3_ solution was added into the reaction mixture, extracted with DCM, and dried over Na_2_SO_4_. After evaporation, the residue was purified by silica gel column chromatography (Wakogel C-300, 0.040–0.063 mm, DCM/Et_2_O = 20/1 to 6.7/1 → DCM/MeOH = 200/1 to 67/1) and recrystallization (from DCM/hexane) to give **1c-Zn** (4.0 mg, 5.2 μmol) in 18% crude yield as a dark green solid. Then, the sample was re-purified by HPLC (Cosmosil 5C_18_-AR-II 10ϕ × 250 mm, MeOH/pyridine = 95/5, 2.0 mL/min) and recrystallization with DCM and hexane: mp > 300 ℃; Vis (THF) λ_max_/nm = 668 (relative intensity, 0.83), 619 (0.11), 571 (0.07), 528 (0.04), 432 (1.00); ^1^H NMR (1.5% pyridine-*d*_5_–CDCl_3_, 600 MHz) δ/ppm = 9.60 (1H, s, 10-H), 9.58 (1H, s, 5-H), 8.402 (1H, s, 20-H), 8.397 (1H, d, *J* = 1 Hz, 1-H of 3^2^-2-naphthyl), 7.98 (1H, d, *J* = 8 Hz, 8-H of 3^2^-2-naphthyl), 7.97 (1H, d, *J* = 8 Hz, 4-H of 3^2^-2-naphthyl), 7.96 (1H, dd, *J* = 8, 1 Hz, 3-H of 3^2^-2-naphthyl), 7.92 (1H, s, 5-H of 3^2^-2-naphthyl), 7.60 (1H, d, *J* = 8 Hz, 7-H of 3^2^-2-naphthyl), 5.20, 5.08 (each 1H, d, *J* = 19 Hz, 13^1^-CH_2_), 4.95 (2H, s, 6-CH_2_ of 3^2^-2-naphthyl), 4.42 (1H, br-q, *J* = 8 Hz, 18-H), 4.23 (1H, br-d, *J* = 8 Hz, 17-H), 3.78 (2H, q, *J* = 8 Hz, 8-CH_2_), 3.70 (3H, s, 12-CH_3_), 3.58 (3H, s, 17^2^-CO_2_CH_3_), 3.51 (3H, s, 2-CH_3_), 3.33 (3H, s, 7-CH_3_), 2.61–2.55, 2.30–2,.24 (each 1H, m, 17-CH_2_), 2.42–2.37, 2.01–1.96 (each 1H, m, 17^1^-CH_2_), 1.74 (3H, d, *J* = 8 Hz, 18-CH_3_), 1.72 (3H, t, *J* = 8 Hz, 8^1^-CH_3_) [The 6-COH signal of 3^2^-2-naphthyl was invisible.]; HRMS (APCI) found: *m*/*z* = 765.2388, calcd for C_45_H_41_N_4_O_4_Zn: MH^+^, 765.2414.

**Self-aggregation of zinc Chl-*a* derivatives in an aqueous TX-100 micellar solution**

Synthetic zinc Chl-*a* derivatives were dissolved in THF (20 μL) containing 2.5% (wt/v) TX-100, and the 1.0 mM solution was diluted 99-fold with distilled water (1980 μL). The solution was allowed to stand in the dark at room temperature. The concentrations of the samples were 10 μM. All optical spectra were measured at room temperature.

**Computational details**

The structure optimization of monomeric Chl-*a* derivative and its *J*-aggregate were carried out by HyperChem Release 8.0.10. The MM+/PM3 calculations were performed until the RMS gradient reached a value below 0.01 kcal Å^−1^ mol^−1^ by using Polak-Ribiere algorithm.

**Reference**

s1. Sasaki S, Mizutani K, Kunieda M, Tamiaki H. Synthesis and optical properties of C3-ethynylated chlorin and π-extended chlorophyll dyads. *Tetrahedron* 2011;67:6065-6072.

**^1^H NMR spectra of Chl-*a* derivatives**

**
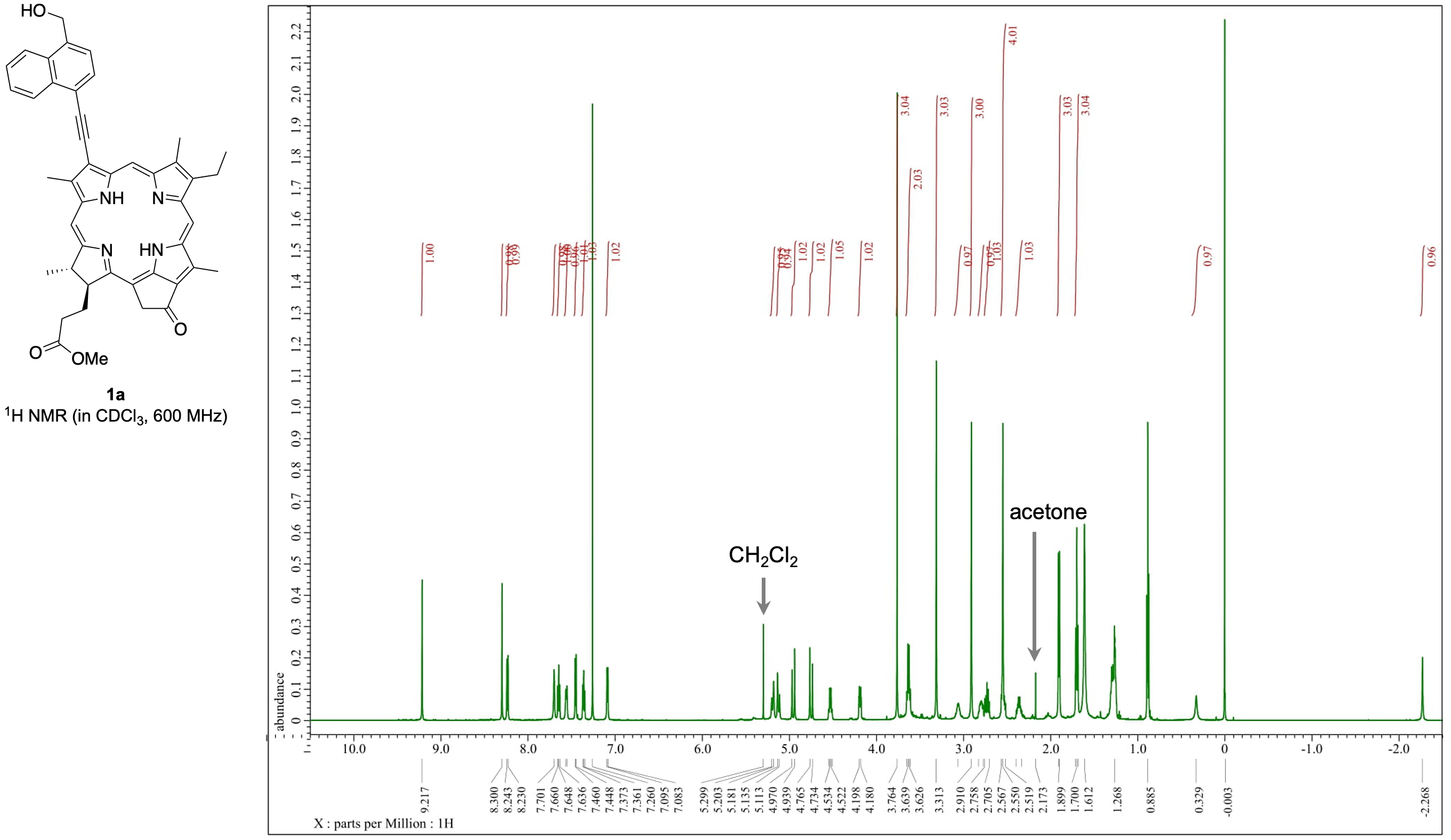
**

**
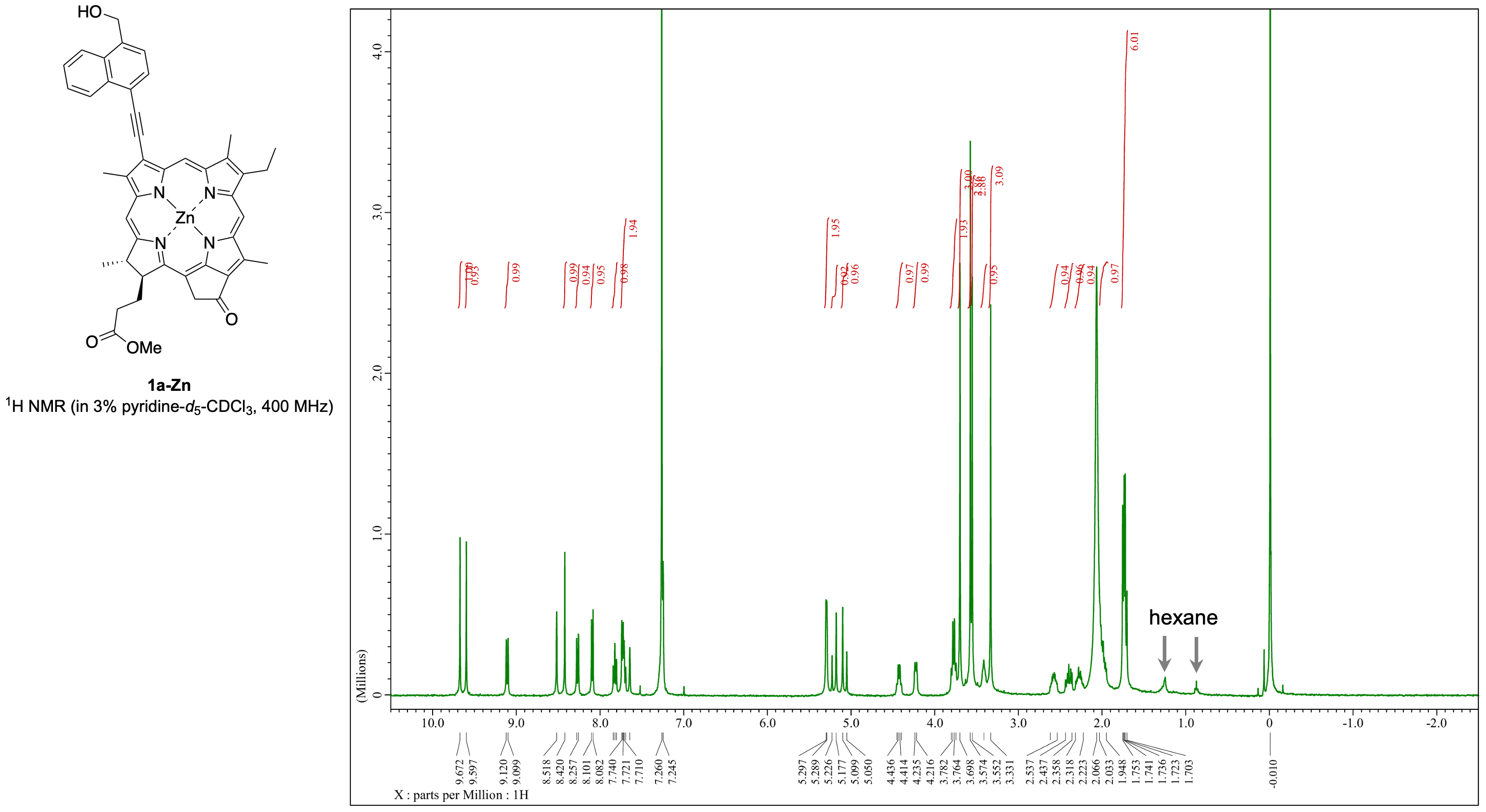
**

**
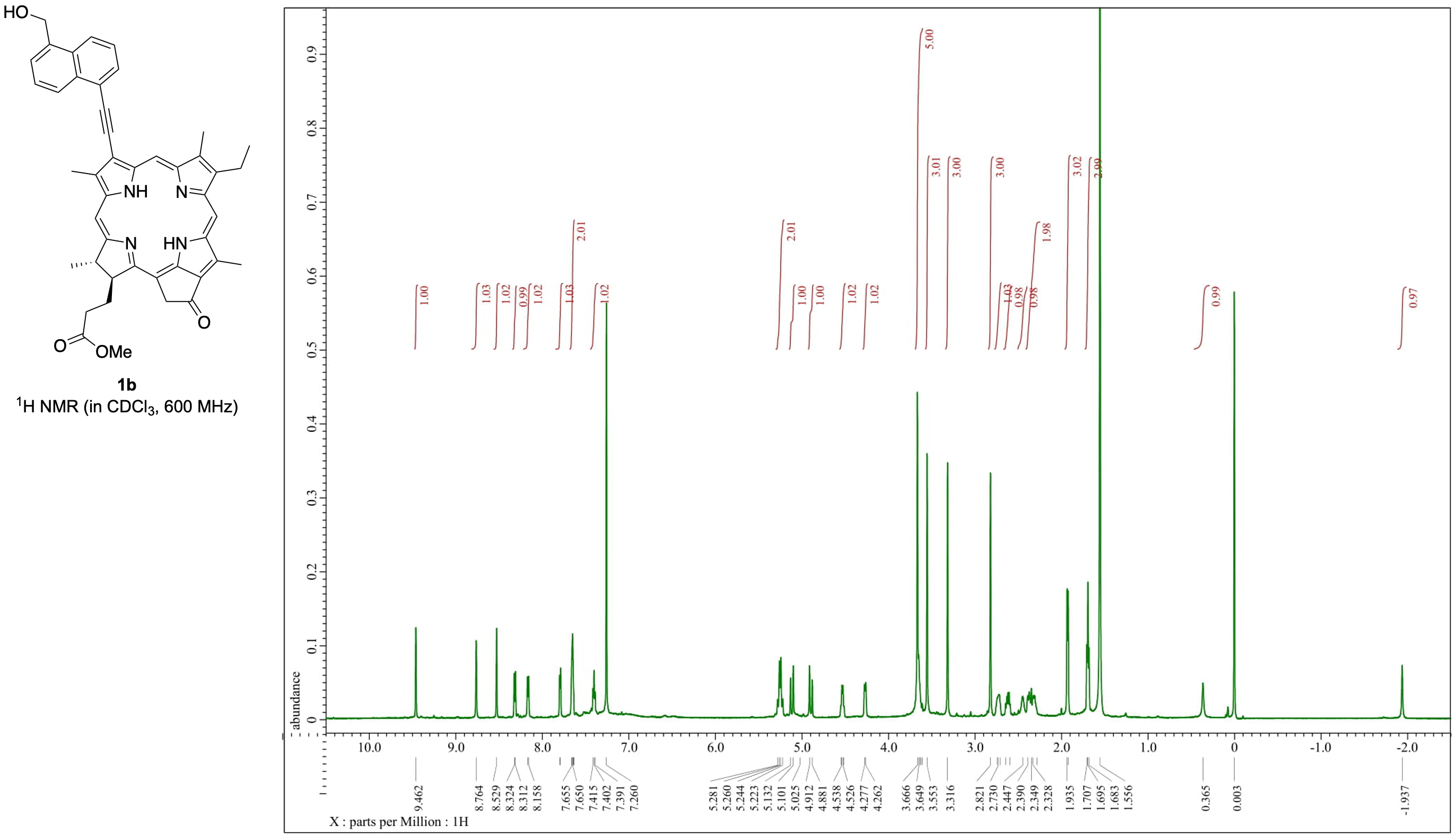
**

**
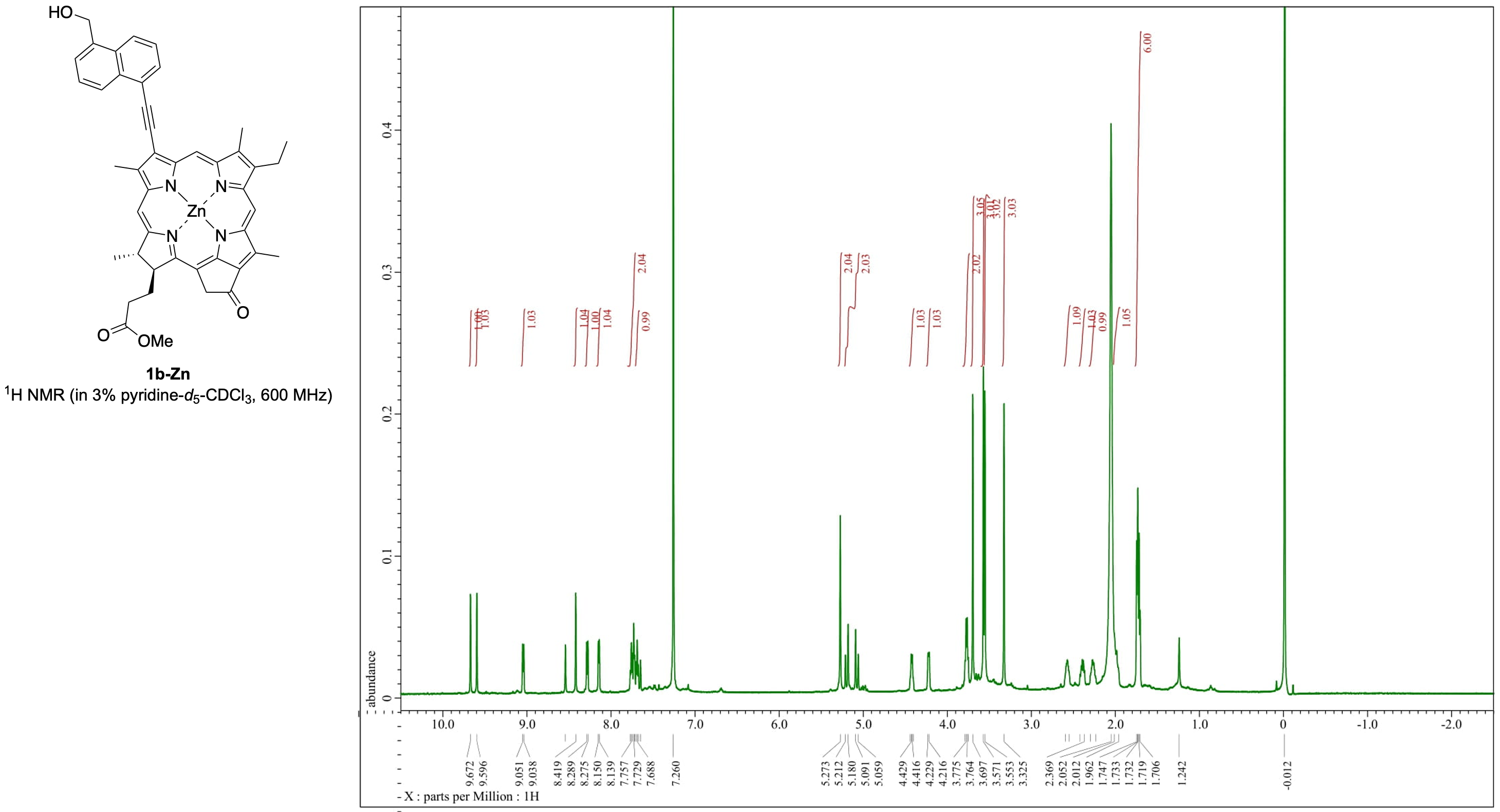
**

**
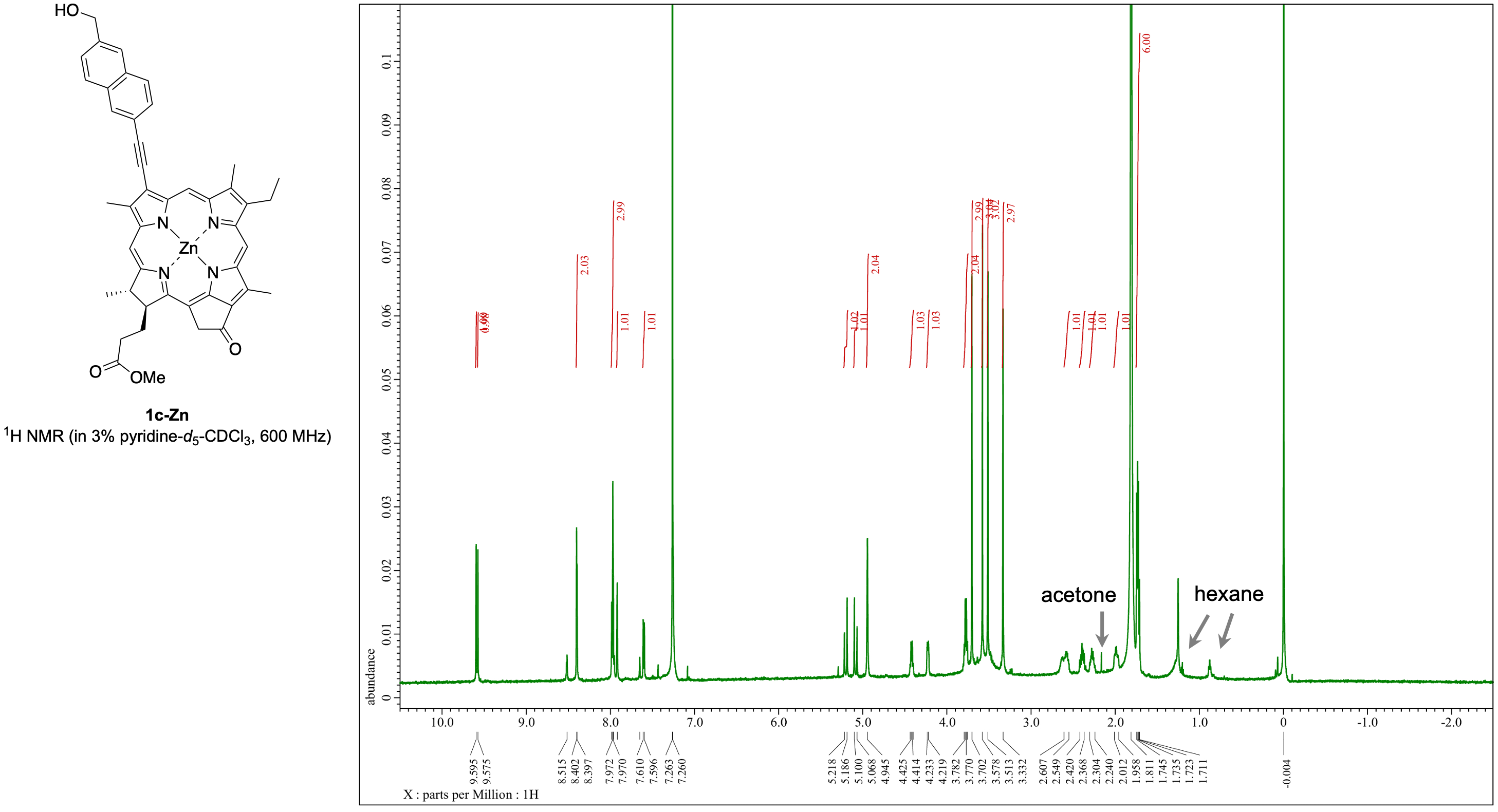
**

**Mass spectra of Chl-*a* derivatives**

**
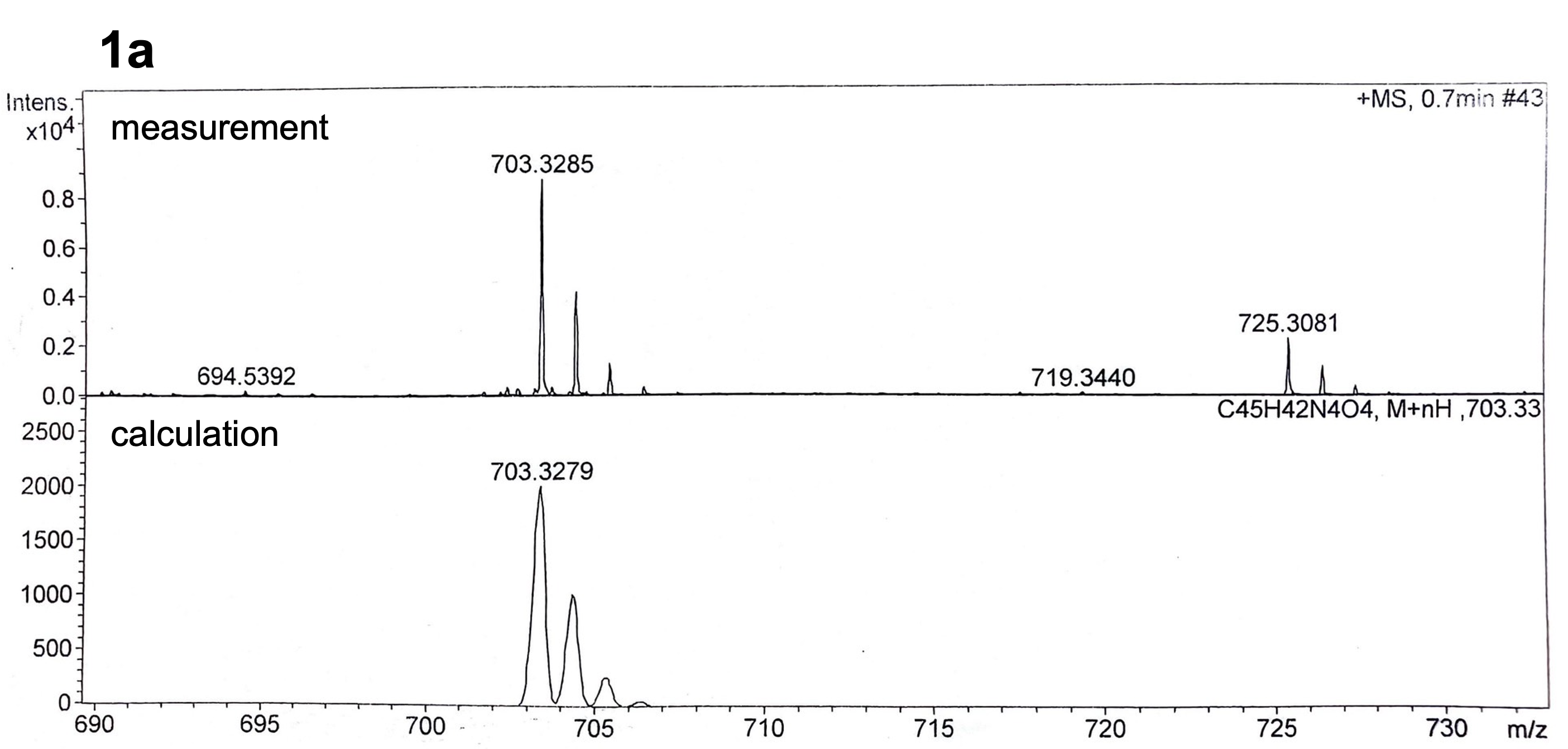
**

**
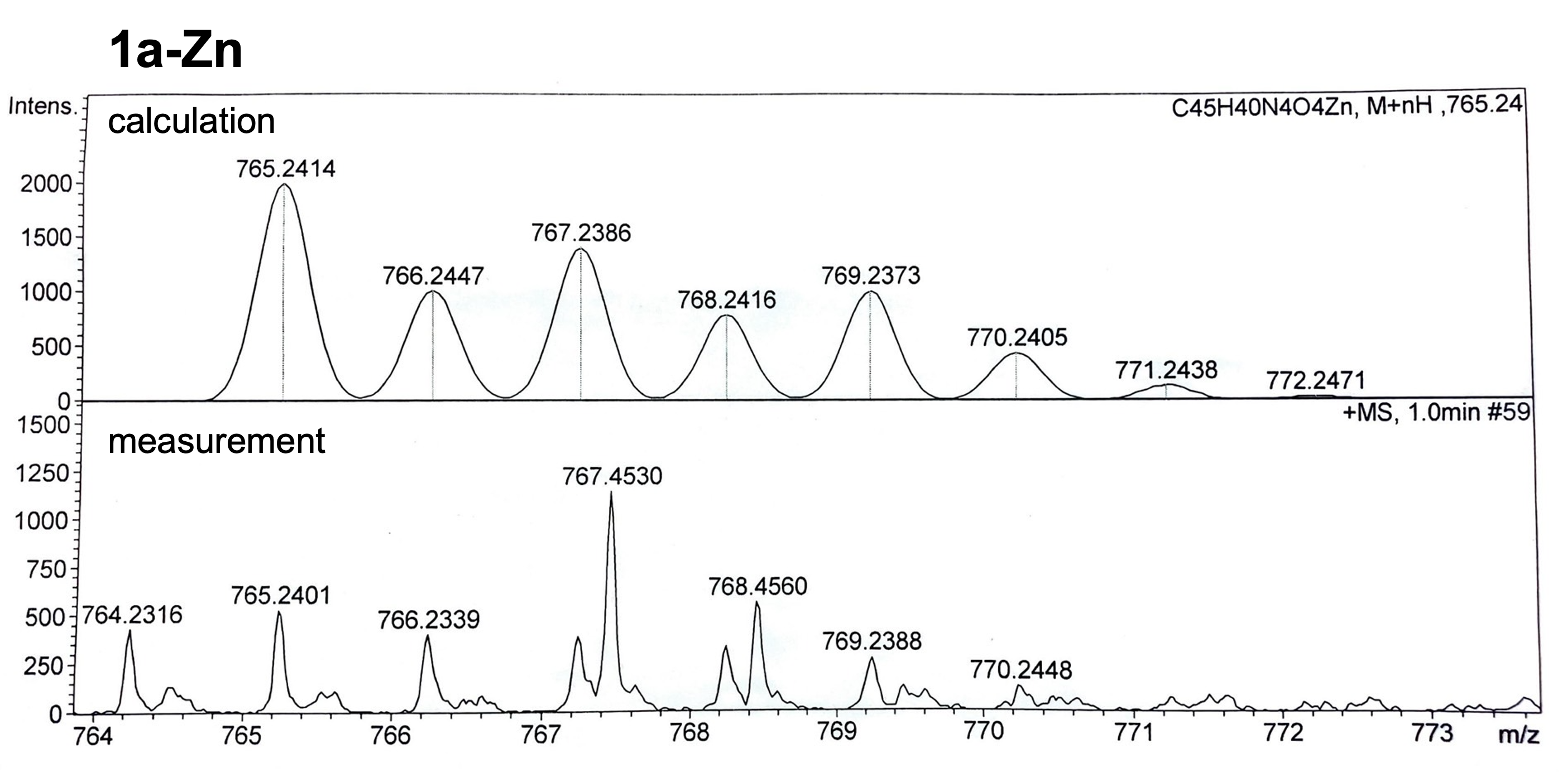
**

**
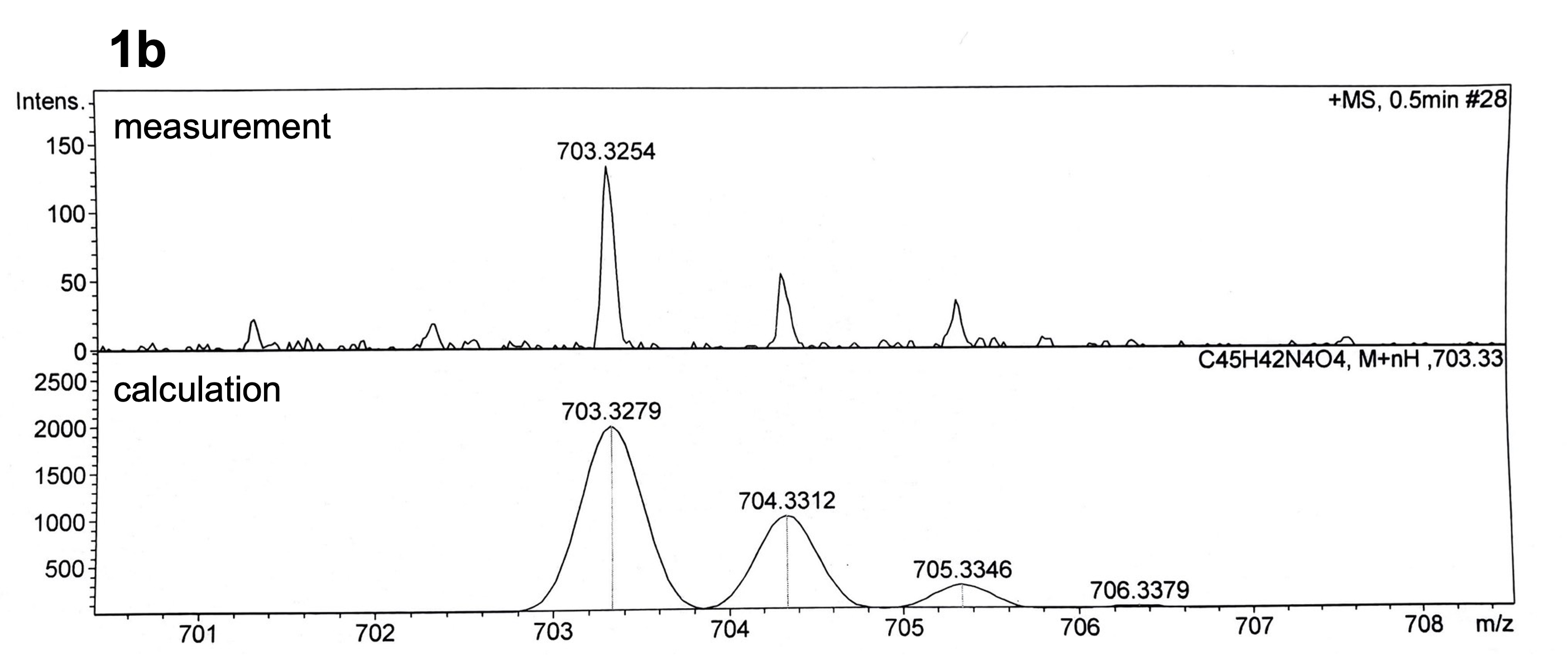
**

**
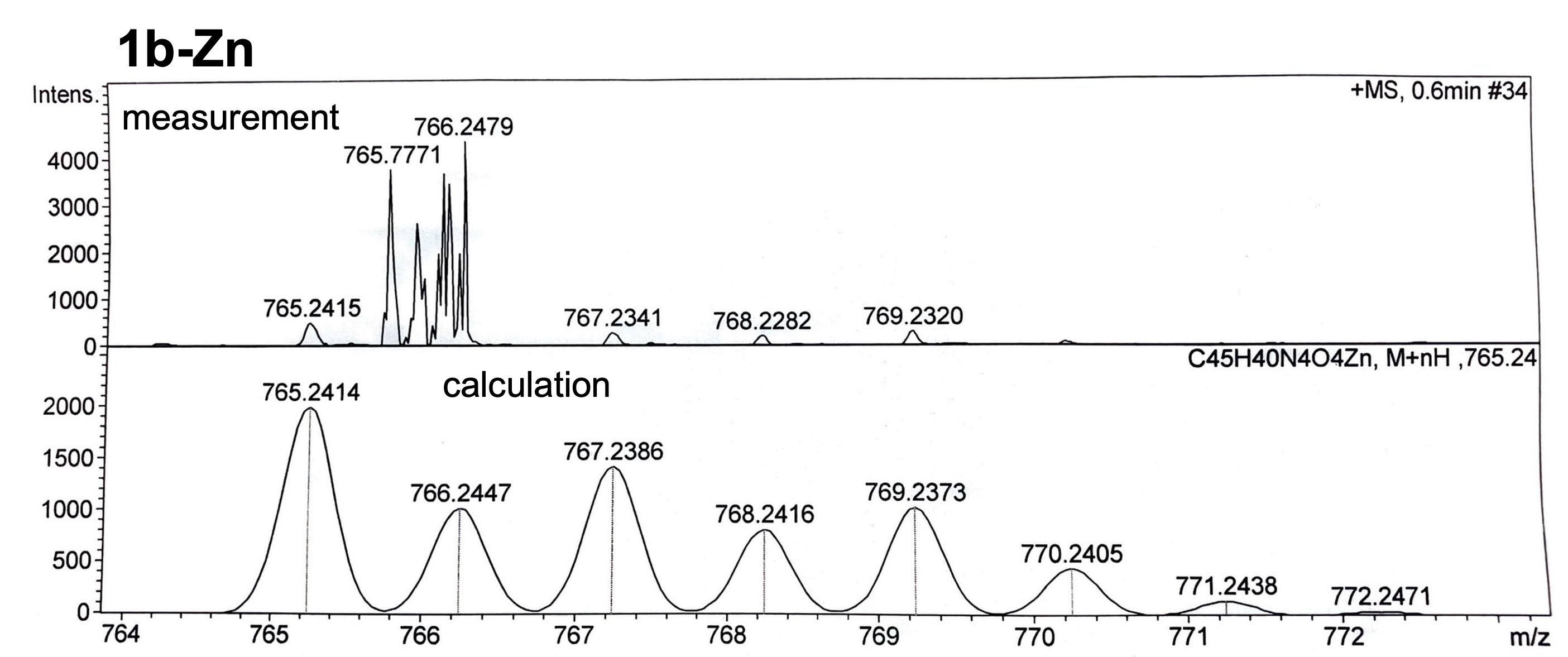
**

**
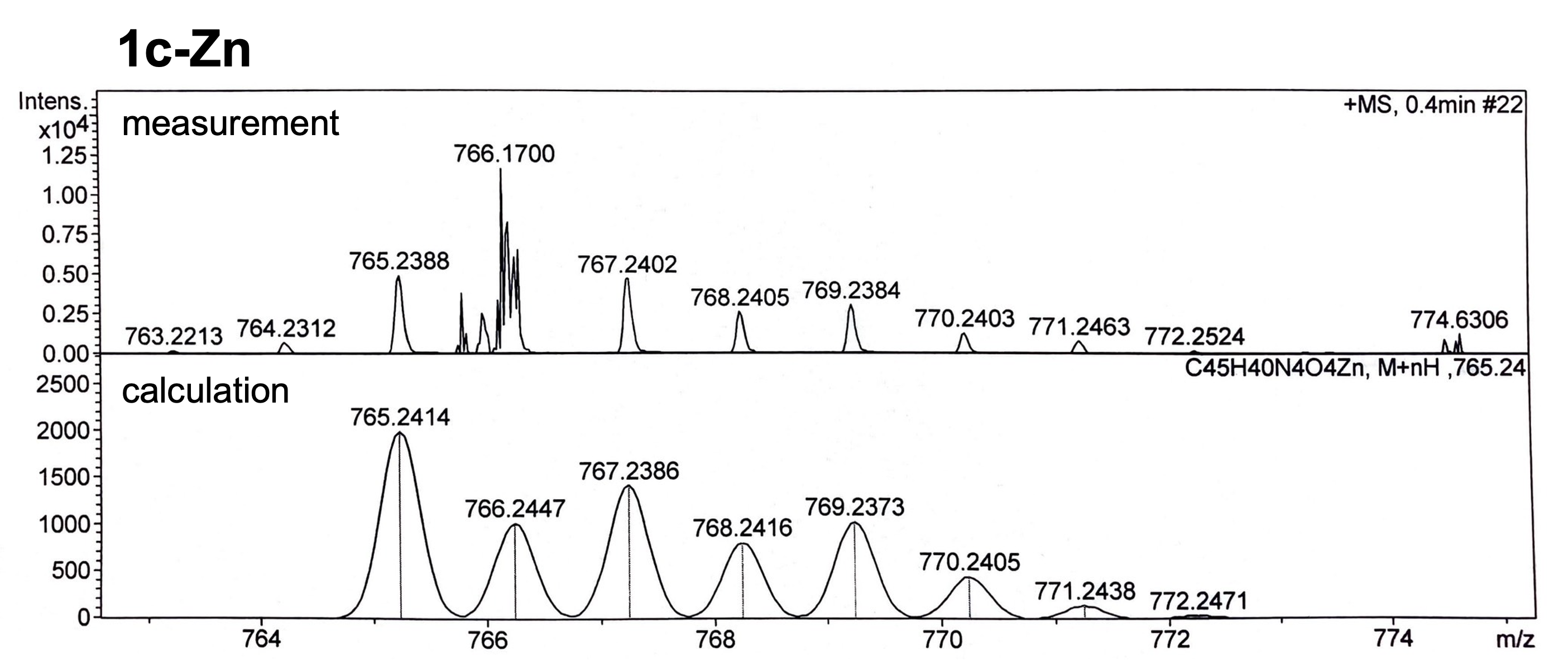
**

**Electronic absorption and CD spectra of 1a-Zn at 20 °C and 55 °C**

**
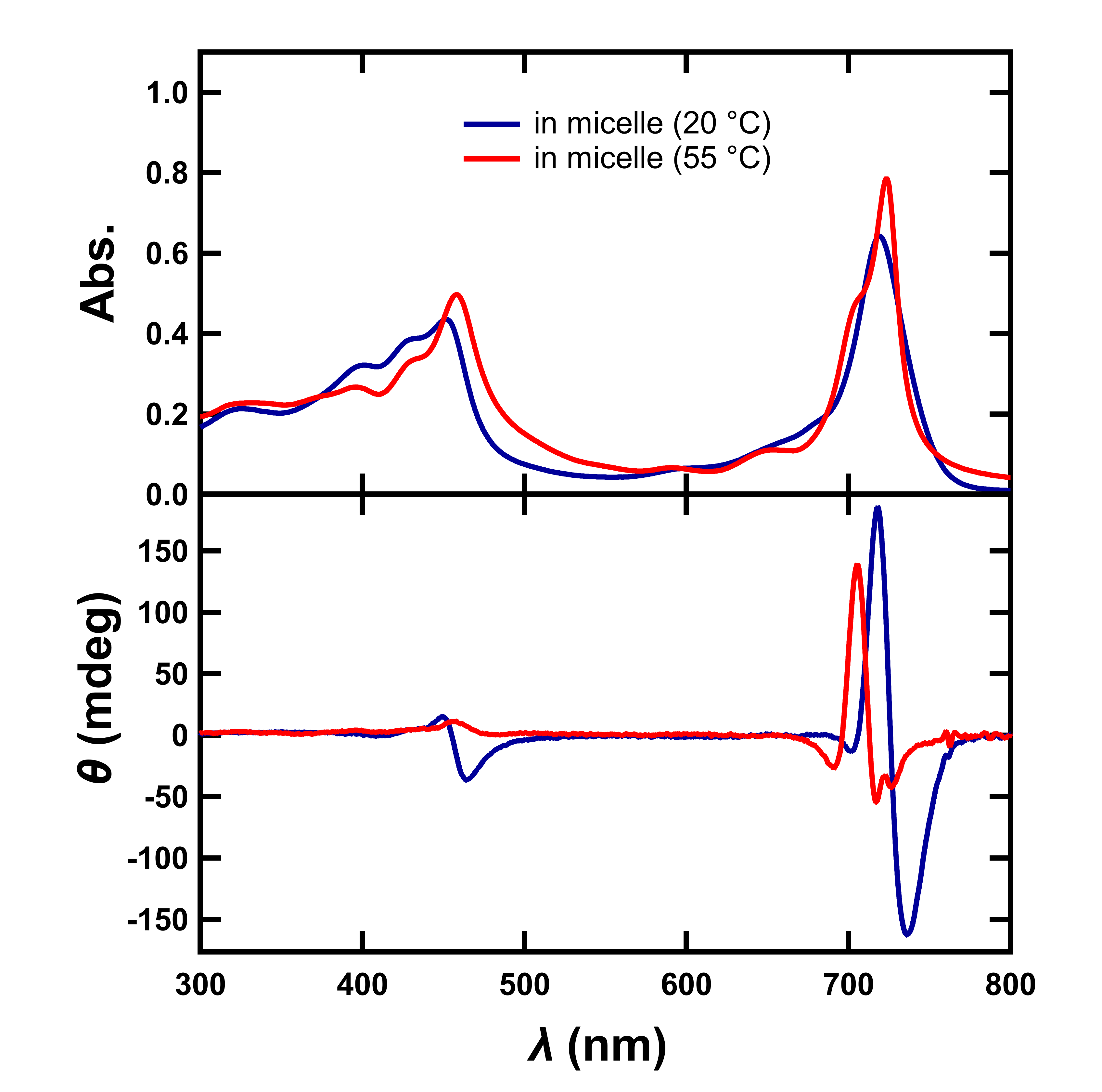
**

**Data of DLS: A and 1c-Zn in micelle**

**
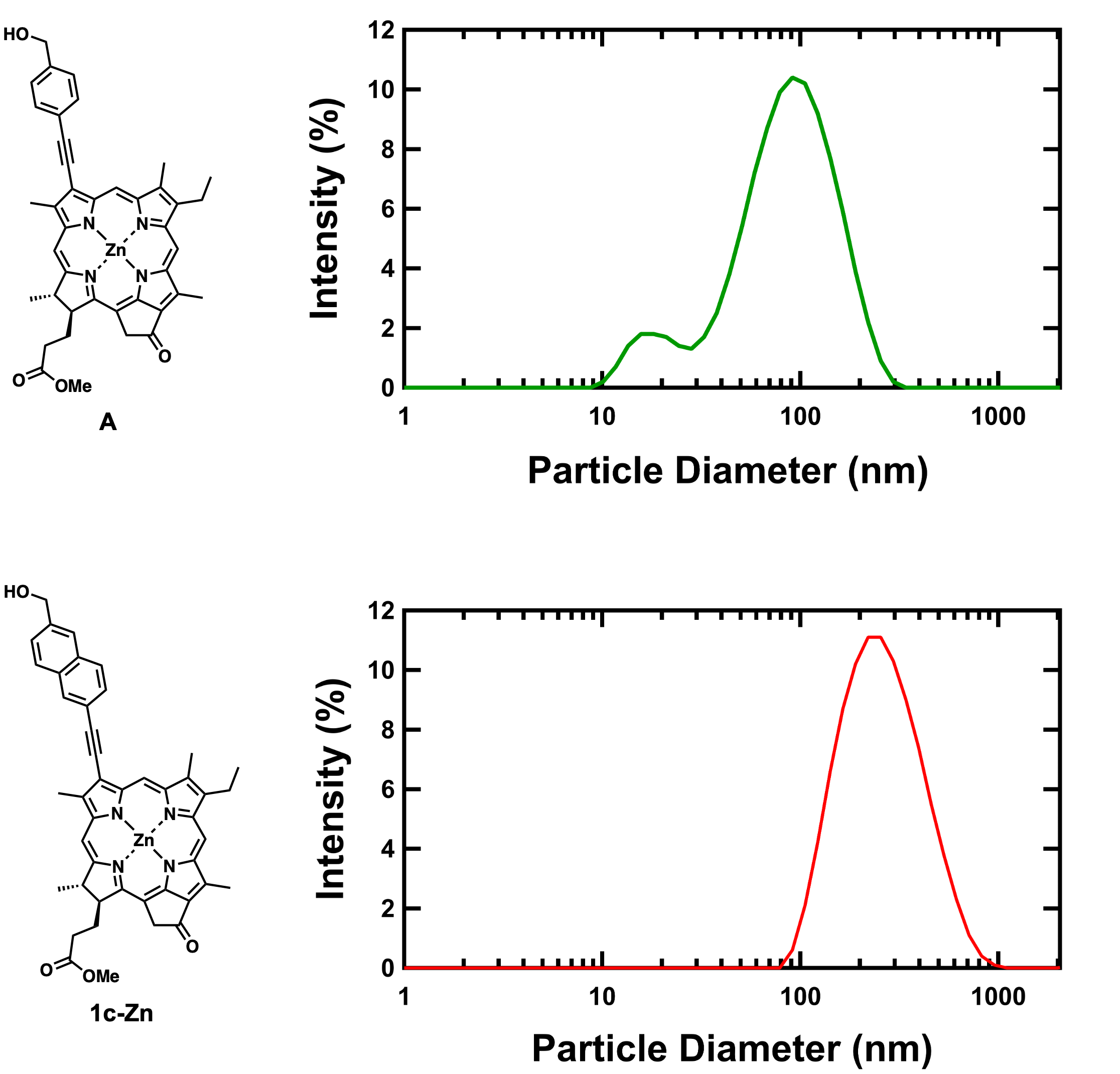
**

**Data of model calculation**

**Energy-minimized structure of 1c-Zn with single α-axial THF (MM+/PM3)**

**
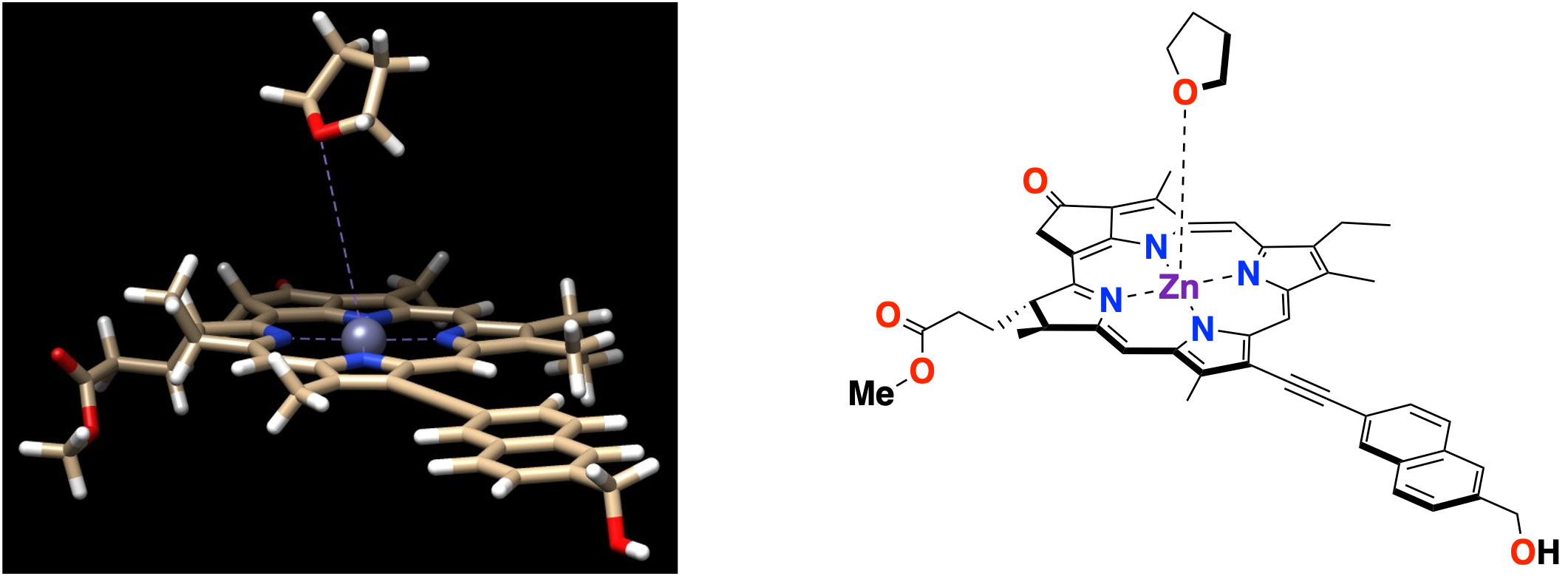
**

107118 0 0 0 0 0 0 0 0999 V2000

65.5640 10.8770 -11.6099 O 0 0 0 0 0 0 0 0 0 0 0 0

64.4025 10.5046 -12.3399 C 0 0 0 0 0 0 0 0 0 0 0 0

63.4955 9.6736 -11.4294 C 0 0 0 0 0 0 0 0 0 0 0 0

64.1029 9.8071 -10.0349 C 0 0 0 0 0 0 0 0 0 0 0 0

63.4549 8.6183 -11.7544 H 0 0 0 0 0 0 0 0 0 0 0 0

62.4521 10.0349 -11.4613 H 0 0 0 0 0 0 0 0 0 0 0 0

63.3880 10.2418 -9.3136 H 0 0 0 0 0 0 0 0 0 0 0 0

64.3833 8.8225 -9.6191 H 0 0 0 0 0 0 0 0 0 0 0 0

64.7770 9.9567 -13.2192 H 0 0 0 0 0 0 0 0 0 0 0 0

63.9139 11.4284 -12.6967 H 0 0 0 0 0 0 0 0 0 0 0 0

67.7668 16.0255 -18.7633 C 0 0 0 0 0 0 0 0 0 0 0 0

69.2282 15.6355 -18.7668 C 0 0 0 0 0 0 0 0 0 0 0 0

70.0830 16.6835 -18.5614 O 0 0 0 0 0 0 0 0 0 0 0 0

69.6922 14.5287 -18.9592 O 0 0 0 0 0 0 0 0 0 0 0 0

67.6100 16.7597 -19.5796 H 0 0 0 0 0 0 0 0 0 0 0 0

67.1577 15.1376 -19.0308 H 0 0 0 0 0 0 0 0 0 0 0 0

71.4693 16.4230 -18.5024 C 0 0 0 0 0 0 0 0 0 0 0 0

71.8481 16.0067 -19.4419 H 0 0 0 0 0 0 0 0 0 0 0 0

71.7170 15.7516 -17.6730 H 0 0 0 0 0 0 0 0 0 0 0 0

68.9437 15.8569 -11.8428 C 0 0 0 0 0 0 0 0 0 0 0 0

67.8187 16.3820 -11.2540 N 0 0 0 0 0 0 0 0 0 0 0 0

68.0904 16.5629 -9.8551 C 0 0 0 0 0 0 0 0 0 0 0 0

69.4794 16.1268 -9.6281 C 0 0 0 0 0 0 0 0 0 0 0 0

67.2275 17.0311 -8.9163 C 0 0 0 0 0 0 0 0 0 0 0 0

65.8846 17.4408 -9.1544 C 0 0 0 0 0 0 0 0 0 0 0 0

65.2410 17.4509 -10.3741 N 0 0 0 0 0 0 0 0 0 0 0 0

63.9323 17.9415 -10.1701 C 0 0 0 0 0 0 0 0 0 0 0 0

63.7801 18.2328 -8.7639 C 0 0 0 0 0 0 0 0 0 0 0 0

64.9784 17.9221 -8.1395 C 0 0 0 0 0 0 0 0 0 0 0 0

62.9454 18.1164 -11.1512 C 0 0 0 0 0 0 0 0 0 0 0 0

63.1129 17.8228 -12.4858 C 0 0 0 0 0 0 0 0 0 0 0 0

64.3353 17.2782 -13.0088 N 0 0 0 0 0 0 0 0 0 0 0 0

64.1317 17.0924 -14.3237 C 0 0 0 0 0 0 0 0 0 0 0 0

62.8019 17.5102 -14.7253 C 0 0 0 0 0 0 0 0 0 0 0 0

62.1601 17.9631 -13.5943 C 0 0 0 0 0 0 0 0 0 0 0 0

64.9048 16.5807 -15.4058 C 0 0 0 0 0 0 0 0 0 0 0 0

66.1801 16.1440 -15.2511 C 0 0 0 0 0 0 0 0 0 0 0 0

66.8457 16.1537 -13.9915 N 0 0 0 0 0 0 0 0 0 0 0 0

68.1108 15.6275 -14.1597 C 0 0 0 0 0 0 0 0 0 0 0 0

68.3570 15.1840 -15.5930 C 0 0 0 0 0 0 0 0 0 0 0 0

67.0632 15.5878 -16.3450 C 0 0 0 0 0 0 0 0 0 0 0 0

69.0859 15.5035 -13.1908 C 0 0 0 0 0 0 0 0 0 0 0 0

70.1212 16.1590 -8.3819 C 0 0 0 0 0 0 0 0 0 0 0 0

67.5800 17.1058 -7.8759 H 0 0 0 0 0 0 0 0 0 0 0 0

62.5710 18.7473 -8.0822 C 0 0 0 0 0 0 0 0 0 0 0 0

61.9728 18.5170 -10.8262 H 0 0 0 0 0 0 0 0 0 0 0 0

62.6896 17.2778 -16.1739 C 0 0 0 0 0 0 0 0 0 0 0 0

60.7958 18.4912 -13.4676 C 0 0 0 0 0 0 0 0 0 0 0 0

64.0342 16.6523 -16.6336 C 0 0 0 0 0 0 0 0 0 0 0 0

69.2400 15.7350 -16.0003 H 0 0 0 0 0 0 0 0 0 0 0 0

66.5680 14.6867 -16.7855 H 0 0 0 0 0 0 0 0 0 0 0 0

70.0555 15.0796 -13.4892 H 0 0 0 0 0 0 0 0 0 0 0 0

69.9970 15.7003 -10.8236 C 0 0 0 0 0 0 0 0 0 0 0 0

62.3376 20.2192 -8.3535 C 0 0 0 0 0 0 0 0 0 0 0 0

68.6231 13.6923 -15.6810 C 0 0 0 0 0 0 0 0 0 0 0 0

62.7005 18.5804 -6.9876 H 0 0 0 0 0 0 0 0 0 0 0 0

61.6873 18.1531 -8.3869 H 0 0 0 0 0 0 0 0 0 0 0 0

61.7511 17.5123 -16.9028 O 0 0 0 0 0 0 0 0 0 0 0 0

60.2661 18.4828 -14.4289 H 0 0 0 0 0 0 0 0 0 0 0 0

60.8117 19.5263 -13.0988 H 0 0 0 0 0 0 0 0 0 0 0 0

60.2118 17.8946 -12.7531 H 0 0 0 0 0 0 0 0 0 0 0 0

63.8683 15.6480 -17.0658 H 0 0 0 0 0 0 0 0 0 0 0 0

64.5289 17.2577 -17.4228 H 0 0 0 0 0 0 0 0 0 0 0 0

62.1738 20.4155 -9.4217 H 0 0 0 0 0 0 0 0 0 0 0 0

63.1944 20.8285 -8.0383 H 0 0 0 0 0 0 0 0 0 0 0 0

61.4553 20.5839 -7.8122 H 0 0 0 0 0 0 0 0 0 0 0 0

71.3433 15.1734 -11.0904 C 0 0 0 0 0 0 0 0 0 0 0 0

71.2918 14.1479 -11.4824 H 0 0 0 0 0 0 0 0 0 0 0 0

71.8641 15.7883 -11.8377 H 0 0 0 0 0 0 0 0 0 0 0 0

67.2852 16.6224 -17.4494 C 0 0 0 0 0 0 0 0 0 0 0 0

67.9747 17.4184 -17.1057 H 0 0 0 0 0 0 0 0 0 0 0 0

66.3101 17.1322 -17.6437 H 0 0 0 0 0 0 0 0 0 0 0 0

67.7875 13.0995 -15.2858 H 0 0 0 0 0 0 0 0 0 0 0 0

69.5197 13.4157 -15.1099 H 0 0 0 0 0 0 0 0 0 0 0 0

68.7849 13.3842 -16.7231 H 0 0 0 0 0 0 0 0 0 0 0 0

71.9560 15.1570 -10.1795 H 0 0 0 0 0 0 0 0 0 0 0 0

65.2606 18.0462 -6.7005 C 0 0 0 0 0 0 0 0 0 0 0 0

71.8876 17.4174 -18.3279 H 0 0 0 0 0 0 0 0 0 0 0 0

65.9971 18.8367 -6.5051 H 0 0 0 0 0 0 0 0 0 0 0 0

64.3339 18.3000 -6.1579 H 0 0 0 0 0 0 0 0 0 0 0 0

65.6554 17.1102 -6.2841 H 0 0 0 0 0 0 0 0 0 0 0 0

70.6756 16.1845 -7.3220 C 0 0 0 0 0 0 0 0 0 0 0 0

71.3330 16.2145 -6.0699 C 0 0 0 0 0 0 0 0 0 0 0 0

72.6546 15.8404 -5.9659 C 0 0 0 0 0 0 0 0 0 0 0 0

73.3034 15.8759 -4.7040 C 0 0 0 0 0 0 0 0 0 0 0 0

72.5858 16.2932 -3.5643 C 0 0 0 0 0 0 0 0 0 0 0 0

71.2225 16.6744 -3.6993 C 0 0 0 0 0 0 0 0 0 0 0 0

70.6092 16.6375 -4.9207 C 0 0 0 0 0 0 0 0 0 0 0 0

73.2181 15.5146 -6.8480 H 0 0 0 0 0 0 0 0 0 0 0 0

70.6740 16.9999 -2.8081 H 0 0 0 0 0 0 0 0 0 0 0 0

69.5597 16.9310 -5.0313 H 0 0 0 0 0 0 0 0 0 0 0 0

66.1050 16.8095 -12.1646 Zn 0 0 0 0 0 0 0 0 0 0 0 0

65.3277 10.7063 -10.2183 C 0 0 0 0 0 0 0 0 0 0 0 0

66.2605 10.2793 -9.8170 H 0 0 0 0 0 0 0 0 0 0 0 0

65.1906 11.7066 -9.7711 H 0 0 0 0 0 0 0 0 0 0 0 0

74.6671 15.4995 -4.5664 C 0 0 0 0 0 0 0 0 0 0 0 0

75.2750 15.5387 -3.3416 C 0 0 0 0 0 0 0 0 0 0 0 0

73.2376 16.3276 -2.3030 C 0 0 0 0 0 0 0 0 0 0 0 0

74.5537 15.9539 -2.1929 C 0 0 0 0 0 0 0 0 0 0 0 0

75.2490 15.9762 -0.8602 C 0 0 0 0 0 0 0 0 0 0 0 0

75.9752 17.1828 -0.7746 O 0 0 0 0 0 0 0 0 0 0 0 0

75.2210 15.1814 -5.4570 H 0 0 0 0 0 0 0 0 0 0 0 0

76.3280 15.2550 -3.2324 H 0 0 0 0 0 0 0 0 0 0 0 0

72.6723 16.6596 -1.4234 H 0 0 0 0 0 0 0 0 0 0 0 0

75.9307 15.1068 -0.7673 H 0 0 0 0 0 0 0 0 0 0 0 0

74.5163 15.9114 -0.0307 H 0 0 0 0 0 0 0 0 0 0 0 0

76.4236 17.1647 0.0600 H 0 0 0 0 0 0 0 0 0 0 0 0

1 2 1 0 0 0 0

1 92 1 0 0 0 0

1 93 1 0 0 0 0

2 3 1 0 0 0 0

2 9 1 0 0 0 0

2 10 1 0 0 0 0

3 4 1 0 0 0 0

3 5 1 0 0 0 0

3 6 1 0 0 0 0

4 7 1 0 0 0 0

4 8 1 0 0 0 0

4 93 1 0 0 0 0

11 12 1 0 0 0 0

11 15 1 0 0 0 0

11 16 1 0 0 0 0

11 70 1 0 0 0 0

12 13 1 0 0 0 0

12 14 2 0 0 0 0

13 17 1 0 0 0 0

17 18 1 0 0 0 0

17 19 1 0 0 0 0

17 78 1 0 0 0 0

20 21 1 0 0 0 0

20 42 1 0 0 0 0

20 53 2 0 0 0 0

21 22 1 0 0 0 0

21 92 1 0 0 0 0

22 23 2 0 0 0 0

22 24 1 0 0 0 0

23 43 1 0 0 0 0

23 53 1 0 0 0 0

24 25 2 0 0 0 0

24 44 1 0 0 0 0

25 26 1 0 0 0 0

25 29 1 0 0 0 0

26 27 2 0 0 0 0

26 92 1 0 0 0 0

27 28 1 0 0 0 0

27 30 1 0 0 0 0

28 29 2 0 0 0 0

28 45 1 0 0 0 0

29 77 1 0 0 0 0

30 31 2 0 0 0 0

30 46 1 0 0 0 0

31 32 1 0 0 0 0

31 35 1 0 0 0 0

32 33 1 0 0 0 0

32 92 1 0 0 0 0

33 34 1 0 0 0 0

33 36 2 0 0 0 0

34 35 2 0 0 0 0

34 47 1 0 0 0 0

35 48 1 0 0 0 0

36 37 1 0 0 0 0

36 49 1 0 0 0 0

37 38 2 0 0 0 0

37 41 1 0 0 0 0

38 39 1 0 0 0 0

38 92 1 0 0 0 0

39 40 1 0 0 0 0

39 42 2 0 0 0 0

40 41 1 0 0 0 0

40 50 1 0 0 0 0

40 55 1 0 0 0 0

41 51 1 0 0 0 0

41 70 1 0 0 0 0

42 52 1 0 0 0 0

43 82 3 0 0 0 0

45 54 1 0 0 0 0

45 56 1 0 0 0 0

45 57 1 0 0 0 0

47 49 1 0 0 0 0

47 58 2 0 0 0 0

48 59 1 0 0 0 0

48 60 1 0 0 0 0

48 61 1 0 0 0 0

49 62 1 0 0 0 0

49 63 1 0 0 0 0

53 67 1 0 0 0 0

54 64 1 0 0 0 0

54 65 1 0 0 0 0

54 66 1 0 0 0 0

55 73 1 0 0 0 0

55 74 1 0 0 0 0

55 75 1 0 0 0 0

67 68 1 0 0 0 0

67 69 1 0 0 0 0

67 76 1 0 0 0 0

70 71 1 0 0 0 0

70 72 1 0 0 0 0

77 79 1 0 0 0 0

77 80 1 0 0 0 0

77 81 1 0 0 0 0

82 83 1 0 0 0 0

83 84 2 0 0 0 0

83 88 1 0 0 0 0

84 85 1 0 0 0 0

84 89 1 0 0 0 0

85 86 2 0 0 0 0

85 96 1 0 0 0 0

86 87 1 0 0 0 0

86 98 1 0 0 0 0

87 88 2 0 0 0 0

87 90 1 0 0 0 0

88 91 1 0 0 0 0

93 94 1 0 0 0 0

93 95 1 0 0 0 0

96 97 2 0 0 0 0

96102 1 0 0 0 0

97 99 1 0 0 0 0

97103 1 0 0 0 0

98 99 2 0 0 0 0

98104 1 0 0 0 0

99100 1 0 0 0 0

100101 1 0 0 0 0

100105 1 0 0 0 0

100106 1 0 0 0 0

101107 1 0 0 0 0

M END

***J*-Aggregate model of 1c-Zn (MM+)**

**
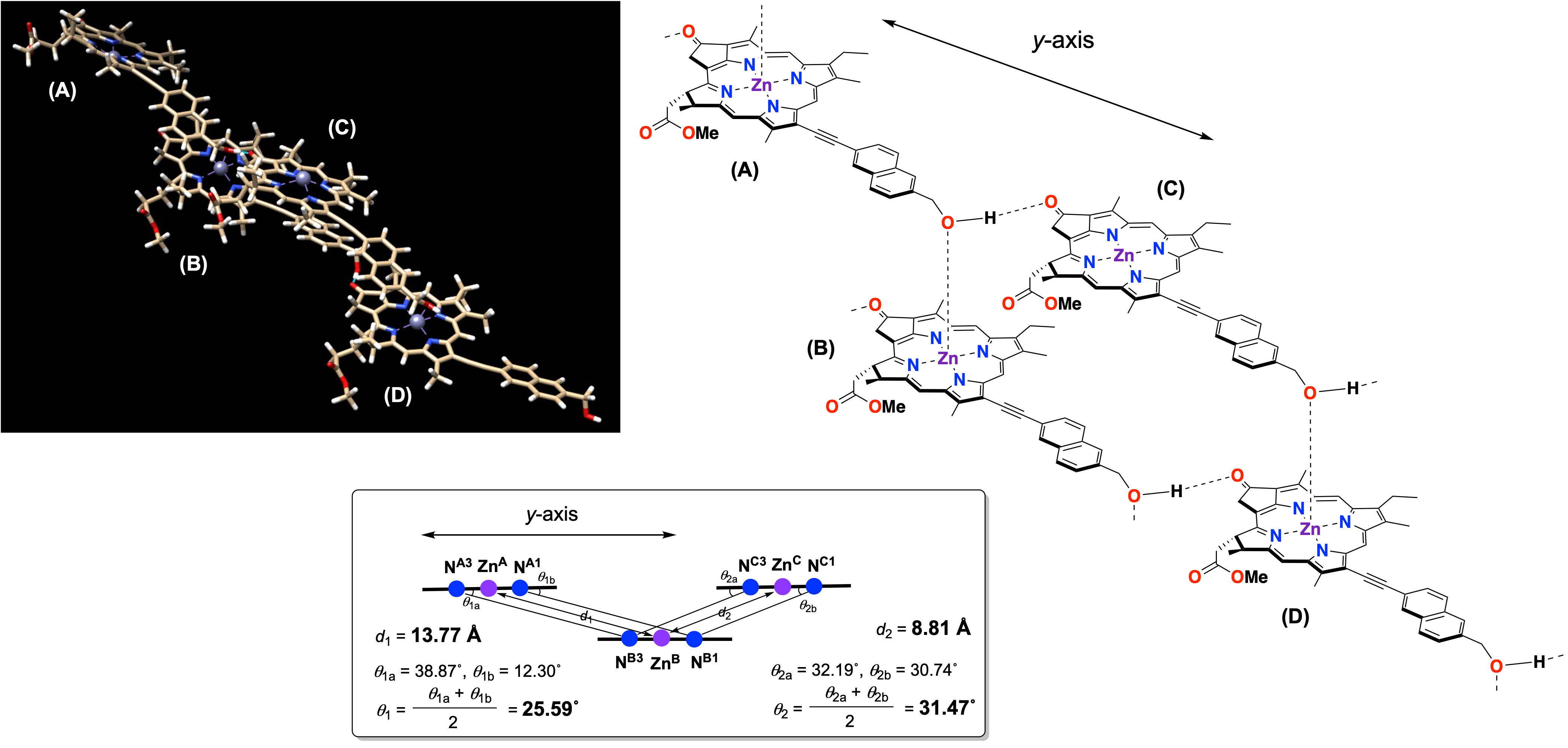
**

376418 0 0 0 0 0 0 0 0999 V2000

384.6946 -14.4342 -33.6736 C 0 0 0 0 0 0 0 0 0 0 0 0

384.1009 -13.2328 -34.3791 C 0 0 0 0 0 0 0 0 0 0 0 0

383.1609 -13.6369 -35.2499 O 0 0 0 0 0 0 0 0 0 0 0 0

384.4337 -12.0854 -34.1955 O 0 0 0 0 0 0 0 0 0 0 0 0

385.0623 -15.1422 -34.4541 H 0 0 0 0 0 0 0 0 0 0 0 0

385.5926 -14.0993 -33.1028 H 0 0 0 0 0 0 0 0 0 0 0 0

382.4987 -12.6359 -35.9826 C 0 0 0 0 0 0 0 0 0 0 0 0

383.2317 -12.1048 -36.6330 H 0 0 0 0 0 0 0 0 0 0 0 0

381.9788 -11.9405 -35.2833 H 0 0 0 0 0 0 0 0 0 0 0 0

378.5982 -14.4021 -30.7972 C 0 0 0 0 0 0 0 0 0 0 0 0

378.6078 -15.3006 -29.8974 N 0 0 0 0 0 0 0 0 0 0 0 0

377.4253 -15.6215 -29.5601 C 0 0 0 0 0 0 0 0 0 0 0 0

376.5848 -14.8795 -30.2974 C 0 0 0 0 0 0 0 0 0 0 0 0

377.0472 -16.5179 -28.6456 C 0 0 0 0 0 0 0 0 0 0 0 0

377.8898 -17.2353 -27.8943 C 0 0 0 0 0 0 0 0 0 0 0 0

379.1578 -17.1855 -27.9024 N 0 0 0 0 0 0 0 0 0 0 0 0

379.5901 -18.0124 -27.0345 C 0 0 0 0 0 0 0 0 0 0 0 0

378.5578 -18.6258 -26.4239 C 0 0 0 0 0 0 0 0 0 0 0 0

377.4529 -18.1295 -26.9919 C 0 0 0 0 0 0 0 0 0 0 0 0

380.8593 -18.3065 -26.7141 C 0 0 0 0 0 0 0 0 0 0 0 0

381.9152 -17.7838 -27.3384 C 0 0 0 0 0 0 0 0 0 0 0 0

381.7282 -16.9088 -28.3482 N 0 0 0 0 0 0 0 0 0 0 0 0

382.9692 -16.6590 -28.7372 C 0 0 0 0 0 0 0 0 0 0 0 0

383.8660 -17.3073 -28.0244 C 0 0 0 0 0 0 0 0 0 0 0 0

383.2275 -18.0433 -27.1120 C 0 0 0 0 0 0 0 0 0 0 0 0

383.4645 -15.8764 -29.6731 C 0 0 0 0 0 0 0 0 0 0 0 0

382.6527 -15.1282 -30.4305 C 0 0 0 0 0 0 0 0 0 0 0 0

381.3819 -15.0357 -30.3265 N 0 0 0 0 0 0 0 0 0 0 0 0

380.9380 -14.1705 -31.1416 C 0 0 0 0 0 0 0 0 0 0 0 0

382.0225 -13.4436 -31.9015 C 0 0 0 0 0 0 0 0 0 0 0 0

383.2418 -14.2890 -31.5425 C 0 0 0 0 0 0 0 0 0 0 0 0

379.6616 -13.8492 -31.3831 C 0 0 0 0 0 0 0 0 0 0 0 0

375.2734 -14.9209 -30.2407 C 0 0 0 0 0 0 0 0 0 0 0 0

375.9644 -16.6620 -28.5022 H 0 0 0 0 0 0 0 0 0 0 0 0

378.5971 -19.6651 -25.3302 C 0 0 0 0 0 0 0 0 0 0 0 0

381.0743 -19.0428 -25.9261 H 0 0 0 0 0 0 0 0 0 0 0 0

385.1314 -17.0362 -28.4079 C 0 0 0 0 0 0 0 0 0 0 0 0

383.8396 -18.9259 -26.0685 C 0 0 0 0 0 0 0 0 0 0 0 0

384.9732 -16.0543 -29.6011 C 0 0 0 0 0 0 0 0 0 0 0 0

381.8025 -13.4873 -32.9940 H 0 0 0 0 0 0 0 0 0 0 0 0

384.0771 -13.6687 -31.1388 H 0 0 0 0 0 0 0 0 0 0 0 0

379.4839 -13.0728 -32.1444 H 0 0 0 0 0 0 0 0 0 0 0 0

377.3217 -14.0911 -31.0879 C 0 0 0 0 0 0 0 0 0 0 0 0

378.7428 -21.0739 -25.9174 C 0 0 0 0 0 0 0 0 0 0 0 0

382.1422 -11.9739 -31.4784 C 0 0 0 0 0 0 0 0 0 0 0 0

377.6728 -19.6088 -24.7110 H 0 0 0 0 0 0 0 0 0 0 0 0

379.4181 -19.4541 -24.6073 H 0 0 0 0 0 0 0 0 0 0 0 0

386.1668 -17.4516 -27.9466 O 0 0 0 0 0 0 0 0 0 0 0 0

384.8595 -18.5686 -25.8112 H 0 0 0 0 0 0 0 0 0 0 0 0

383.9128 -19.9715 -26.4432 H 0 0 0 0 0 0 0 0 0 0 0 0

383.2531 -18.9210 -25.1230 H 0 0 0 0 0 0 0 0 0 0 0 0

385.4908 -15.0916 -29.3850 H 0 0 0 0 0 0 0 0 0 0 0 0

385.3935 -16.5313 -30.5148 H 0 0 0 0 0 0 0 0 0 0 0 0

379.6854 -21.1734 -26.5036 H 0 0 0 0 0 0 0 0 0 0 0 0

377.8914 -21.3150 -26.5953 H 0 0 0 0 0 0 0 0 0 0 0 0

378.7631 -21.8437 -25.1115 H 0 0 0 0 0 0 0 0 0 0 0 0

376.8208 -13.0871 -32.0871 C 0 0 0 0 0 0 0 0 0 0 0 0

377.1861 -12.0672 -31.8287 H 0 0 0 0 0 0 0 0 0 0 0 0

377.1726 -13.3482 -33.1110 H 0 0 0 0 0 0 0 0 0 0 0 0

383.7288 -15.1581 -32.7203 C 0 0 0 0 0 0 0 0 0 0 0 0

382.8613 -15.5780 -33.2817 H 0 0 0 0 0 0 0 0 0 0 0 0

384.2741 -16.0469 -32.3304 H 0 0 0 0 0 0 0 0 0 0 0 0

382.2836 -11.8739 -30.3803 H 0 0 0 0 0 0 0 0 0 0 0 0

381.2315 -11.3934 -31.7516 H 0 0 0 0 0 0 0 0 0 0 0 0

383.0081 -11.4842 -31.9786 H 0 0 0 0 0 0 0 0 0 0 0 0

375.7087 -13.0453 -32.1167 H 0 0 0 0 0 0 0 0 0 0 0 0

376.0262 -18.5055 -26.6935 C 0 0 0 0 0 0 0 0 0 0 0 0

381.7441 -13.1500 -36.6190 H 0 0 0 0 0 0 0 0 0 0 0 0

375.4754 -18.7509 -27.6297 H 0 0 0 0 0 0 0 0 0 0 0 0

375.9406 -19.4081 -26.0500 H 0 0 0 0 0 0 0 0 0 0 0 0

375.5070 -17.6674 -26.1757 H 0 0 0 0 0 0 0 0 0 0 0 0

374.0675 -15.0048 -30.1424 C 0 0 0 0 0 0 0 0 0 0 0 0

372.7672 -15.1368 -29.9890 C 0 0 0 0 0 0 0 0 0 0 0 0

371.9266 -14.1751 -30.4035 C 0 0 0 0 0 0 0 0 0 0 0 0

370.5978 -14.3080 -30.2339 C 0 0 0 0 0 0 0 0 0 0 0 0

370.1065 -15.4162 -29.6447 C 0 0 0 0 0 0 0 0 0 0 0 0

370.9528 -16.3786 -29.2353 C 0 0 0 0 0 0 0 0 0 0 0 0

372.2766 -16.2409 -29.4043 C 0 0 0 0 0 0 0 0 0 0 0 0

372.3397 -13.2695 -30.8823 H 0 0 0 0 0 0 0 0 0 0 0 0

370.5766 -17.2957 -28.7484 H 0 0 0 0 0 0 0 0 0 0 0 0

372.9549 -17.0400 -29.0550 H 0 0 0 0 0 0 0 0 0 0 0 0

380.2085 -15.9343 -28.9992 Zn 0 0 0 0 0 0 0 0 0 0 0 0

369.7459 -13.3496 -30.6393 C 0 0 0 0 0 0 0 0 0 0 0 0

368.7783 -15.5431 -29.4709 C 0 0 0 0 0 0 0 0 0 0 0 0

368.4235 -13.4888 -30.4599 C 0 0 0 0 0 0 0 0 0 0 0 0

367.9220 -14.5878 -29.8724 C 0 0 0 0 0 0 0 0 0 0 0 0

366.4364 -14.7489 -29.6596 C 0 0 0 0 0 0 0 0 0 0 0 0

365.8938 -15.5470 -30.6765 O 0 0 0 0 0 0 0 0 0 0 0 0

370.1180 -12.4300 -31.1244 H 0 0 0 0 0 0 0 0 0 0 0 0

368.3659 -16.4460 -28.9887 H 0 0 0 0 0 0 0 0 0 0 0 0

367.7453 -12.6881 -30.8007 H 0 0 0 0 0 0 0 0 0 0 0 0

365.8945 -13.7759 -29.6854 H 0 0 0 0 0 0 0 0 0 0 0 0

366.2112 -15.2520 -28.6912 H 0 0 0 0 0 0 0 0 0 0 0 0

379.8521 -14.7682 -27.5058 O 0 0 0 0 0 0 0 0 0 0 0 0

398.2502 -16.4527 -25.3145 C 0 0 0 0 0 0 0 0 0 0 0 0

397.9686 -15.0442 -25.7945 C 0 0 0 0 0 0 0 0 0 0 0 0

397.2341 -15.0895 -26.9190 O 0 0 0 0 0 0 0 0 0 0 0 0

398.3437 -14.0345 -25.2460 O 0 0 0 0 0 0 0 0 0 0 0 0

398.7006 -17.0198 -26.1625 H 0 0 0 0 0 0 0 0 0 0 0 0

399.0335 -16.4132 -24.5211 H 0 0 0 0 0 0 0 0 0 0 0 0

396.8436 -13.8584 -27.4746 C 0 0 0 0 0 0 0 0 0 0 0 0

397.7472 -13.2736 -27.7643 H 0 0 0 0 0 0 0 0 0 0 0 0

396.2048 -13.3067 -26.7465 H 0 0 0 0 0 0 0 0 0 0 0 0

391.8567 -16.0242 -23.7592 C 0 0 0 0 0 0 0 0 0 0 0 0

391.5713 -17.1080 -23.1481 N 0 0 0 0 0 0 0 0 0 0 0 0

390.3031 -17.2365 -23.1932 C 0 0 0 0 0 0 0 0 0 0 0 0

389.7590 -16.2271 -23.8887 C 0 0 0 0 0 0 0 0 0 0 0 0

389.5639 -18.2042 -22.6592 C 0 0 0 0 0 0 0 0 0 0 0 0

390.1149 -19.1951 -21.9615 C 0 0 0 0 0 0 0 0 0 0 0 0

391.3476 -19.3274 -21.6841 N 0 0 0 0 0 0 0 0 0 0 0 0

391.4182 -20.4105 -21.0111 C 0 0 0 0 0 0 0 0 0 0 0 0

390.2147 -20.9892 -20.8405 C 0 0 0 0 0 0 0 0 0 0 0 0

389.3547 -20.1883 -21.4717 C 0 0 0 0 0 0 0 0 0 0 0 0

392.5025 -20.9856 -20.4819 C 0 0 0 0 0 0 0 0 0 0 0 0

393.7166 -20.5136 -20.7457 C 0 0 0 0 0 0 0 0 0 0 0 0

393.8489 -19.4405 -21.5638 N 0 0 0 0 0 0 0 0 0 0 0 0

395.1747 -19.3561 -21.5434 C 0 0 0 0 0 0 0 0 0 0 0 0

395.8028 -20.2950 -20.8660 C 0 0 0 0 0 0 0 0 0 0 0 0

394.8745 -21.0781 -20.3248 C 0 0 0 0 0 0 0 0 0 0 0 0

396.0065 -18.4977 -22.0853 C 0 0 0 0 0 0 0 0 0 0 0 0

395.4821 -17.4828 -22.7684 C 0 0 0 0 0 0 0 0 0 0 0 0

394.2389 -17.1926 -22.8576 N 0 0 0 0 0 0 0 0 0 0 0 0

394.1716 -16.0885 -23.4795 C 0 0 0 0 0 0 0 0 0 0 0 0

395.4897 -15.3986 -23.7319 C 0 0 0 0 0 0 0 0 0 0 0 0

396.4372 -16.5607 -23.4912 C 0 0 0 0 0 0 0 0 0 0 0 0

393.0655 -15.4948 -23.9225 C 0 0 0 0 0 0 0 0 0 0 0 0

388.4903 -16.0290 -24.1633 C 0 0 0 0 0 0 0 0 0 0 0 0

388.4733 -18.1777 -22.8122 H 0 0 0 0 0 0 0 0 0 0 0 0

389.8538 -22.2381 -20.0729 C 0 0 0 0 0 0 0 0 0 0 0 0

392.4326 -21.9037 -19.8797 H 0 0 0 0 0 0 0 0 0 0 0 0

397.1481 -20.1945 -20.9002 C 0 0 0 0 0 0 0 0 0 0 0 0

395.0712 -22.2807 -19.4546 C 0 0 0 0 0 0 0 0 0 0 0 0

397.4198 -18.9500 -21.7784 C 0 0 0 0 0 0 0 0 0 0 0 0

395.5492 -15.0260 -24.7814 H 0 0 0 0 0 0 0 0 0 0 0 0

397.2718 -16.2677 -22.8106 H 0 0 0 0 0 0 0 0 0 0 0 0

393.1790 -14.5388 -24.4592 H 0 0 0 0 0 0 0 0 0 0 0 0

390.7601 -15.4293 -24.2591 C 0 0 0 0 0 0 0 0 0 0 0 0

390.0082 -23.4973 -20.9309 C 0 0 0 0 0 0 0 0 0 0 0 0

395.7028 -14.2315 -22.7578 C 0 0 0 0 0 0 0 0 0 0 0 0

388.8059 -22.1652 -19.7011 H 0 0 0 0 0 0 0 0 0 0 0 0

390.4580 -22.3226 -19.1408 H 0 0 0 0 0 0 0 0 0 0 0 0

397.9746 -20.9036 -20.3797 O 0 0 0 0 0 0 0 0 0 0 0 0

396.1399 -22.4088 -19.1740 H 0 0 0 0 0 0 0 0 0 0 0 0

394.7373 -23.1986 -19.9888 H 0 0 0 0 0 0 0 0 0 0 0 0

394.4915 -22.1840 -18.5090 H 0 0 0 0 0 0 0 0 0 0 0 0

397.9796 -18.1812 -21.1979 H 0 0 0 0 0 0 0 0 0 0 0 0

397.9830 -19.2500 -22.6907 H 0 0 0 0 0 0 0 0 0 0 0 0

391.0587 -23.6204 -21.2817 H 0 0 0 0 0 0 0 0 0 0 0 0

389.3421 -23.4536 -21.8232 H 0 0 0 0 0 0 0 0 0 0 0 0

389.7363 -24.4103 -20.3524 H 0 0 0 0 0 0 0 0 0 0 0 0

390.6571 -14.1581 -25.0540 C 0 0 0 0 0 0 0 0 0 0 0 0

391.0431 -13.2963 -24.4638 H 0 0 0 0 0 0 0 0 0 0 0 0

391.2385 -14.2420 -26.0002 H 0 0 0 0 0 0 0 0 0 0 0 0

397.0081 -17.1839 -24.7757 C 0 0 0 0 0 0 0 0 0 0 0 0

396.2154 -17.2596 -25.5570 H 0 0 0 0 0 0 0 0 0 0 0 0

397.3216 -18.2281 -24.5522 H 0 0 0 0 0 0 0 0 0 0 0 0

395.6339 -14.5675 -21.6976 H 0 0 0 0 0 0 0 0 0 0 0 0

394.9433 -13.4303 -22.9095 H 0 0 0 0 0 0 0 0 0 0 0 0

396.7062 -13.7708 -22.9066 H 0 0 0 0 0 0 0 0 0 0 0 0

389.6059 -13.9217 -25.3346 H 0 0 0 0 0 0 0 0 0 0 0 0

387.8682 -20.3854 -21.5991 C 0 0 0 0 0 0 0 0 0 0 0 0

396.2461 -14.0898 -28.3847 H 0 0 0 0 0 0 0 0 0 0 0 0

387.3322 -19.7335 -20.8726 H 0 0 0 0 0 0 0 0 0 0 0 0

387.5027 -20.1547 -22.6231 H 0 0 0 0 0 0 0 0 0 0 0 0

387.5644 -21.4392 -21.4141 H 0 0 0 0 0 0 0 0 0 0 0 0

387.3321 -15.8170 -24.4526 C 0 0 0 0 0 0 0 0 0 0 0 0

386.0937 -15.5525 -24.8112 C 0 0 0 0 0 0 0 0 0 0 0 0

385.8661 -14.6837 -25.8090 C 0 0 0 0 0 0 0 0 0 0 0 0

384.6105 -14.4063 -26.2029 C 0 0 0 0 0 0 0 0 0 0 0 0

383.5680 -15.0029 -25.5925 C 0 0 0 0 0 0 0 0 0 0 0 0

383.8030 -15.8623 -24.5837 C 0 0 0 0 0 0 0 0 0 0 0 0

385.0574 -16.1403 -24.1944 C 0 0 0 0 0 0 0 0 0 0 0 0

386.7253 -14.2046 -26.3118 H 0 0 0 0 0 0 0 0 0 0 0 0

384.3795 -13.5448 -27.2081 C 0 0 0 0 0 0 0 0 0 0 0 0

382.9674 -16.3677 -24.0681 H 0 0 0 0 0 0 0 0 0 0 0 0

385.2278 -16.8593 -23.3734 H 0 0 0 0 0 0 0 0 0 0 0 0

392.7088 -17.8877 -21.7523 Zn 0 0 0 0 0 0 0 0 0 0 0 0

382.3160 -14.7401 -26.0115 C 0 0 0 0 0 0 0 0 0 0 0 0

383.1271 -13.2988 -27.6167 C 0 0 0 0 0 0 0 0 0 0 0 0

382.0773 -13.9022 -27.0359 C 0 0 0 0 0 0 0 0 0 0 0 0

385.2124 -13.0357 -27.7241 H 0 0 0 0 0 0 0 0 0 0 0 0

381.4568 -15.2286 -25.5213 H 0 0 0 0 0 0 0 0 0 0 0 0

382.9673 -12.5942 -28.4472 H 0 0 0 0 0 0 0 0 0 0 0 0

380.6725 -13.6277 -27.5197 C 0 0 0 0 0 0 0 0 0 0 0 0

380.6252 -13.1660 -28.5320 H 0 0 0 0 0 0 0 0 0 0 0 0

380.2050 -12.9112 -26.8063 H 0 0 0 0 0 0 0 0 0 0 0 0

378.9683 -14.4828 -27.6629 H 0 0 0 0 0 0 0 0 0 0 0 0

364.9726 -15.6430 -30.5049 H 0 0 0 0 0 0 0 0 0 0 0 0

400.5523 -20.7800 -22.1132 O 0 0 0 0 0 0 0 0 0 0 0 0

419.1604 -21.4682 -19.1295 C 0 0 0 0 0 0 0 0 0 0 0 0

418.9103 -19.9743 -19.1520 C 0 0 0 0 0 0 0 0 0 0 0 0

418.3331 -19.6282 -20.3153 O 0 0 0 0 0 0 0 0 0 0 0 0

419.1857 -19.2108 -18.2562 O 0 0 0 0 0 0 0 0 0 0 0 0

419.7213 -21.7398 -20.0555 H 0 0 0 0 0 0 0 0 0 0 0 0

419.8355 -21.6999 -18.2722 H 0 0 0 0 0 0 0 0 0 0 0 0

417.9912 -18.2739 -20.4785 C 0 0 0 0 0 0 0 0 0 0 0 0

418.9140 -17.6495 -20.4495 H 0 0 0 0 0 0 0 0 0 0 0 0

417.2597 -17.9753 -19.6921 H 0 0 0 0 0 0 0 0 0 0 0 0

412.5692 -21.3674 -18.4208 C 0 0 0 0 0 0 0 0 0 0 0 0

412.1873 -22.5329 -18.0692 N 0 0 0 0 0 0 0 0 0 0 0 0

410.9270 -22.5779 -18.2548 C 0 0 0 0 0 0 0 0 0 0 0 0

410.4832 -21.4093 -18.7400 C 0 0 0 0 0 0 0 0 0 0 0 0

410.1083 -23.5870 -17.9690 C 0 0 0 0 0 0 0 0 0 0 0 0

410.5680 -24.7077 -17.4154 C 0 0 0 0 0 0 0 0 0 0 0 0

411.7855 -24.9976 -17.1954 N 0 0 0 0 0 0 0 0 0 0 0 0

411.7496 -26.1402 -16.6268 C 0 0 0 0 0 0 0 0 0 0 0 0

410.4913 -26.5794 -16.4319 C 0 0 0 0 0 0 0 0 0 0 0 0

409.7117 -25.6476 -16.9836 C 0 0 0 0 0 0 0 0 0 0 0 0

412.7736 -26.9183 -16.2650 C 0 0 0 0 0 0 0 0 0 0 0 0

414.0264 -26.4875 -16.3648 C 0 0 0 0 0 0 0 0 0 0 0 0

414.2671 -25.2220 -16.7892 N 0 0 0 0 0 0 0 0 0 0 0 0

415.5948 -25.2668 -16.7494 C 0 0 0 0 0 0 0 0 0 0 0 0

416.1218 -26.3720 -16.2646 C 0 0 0 0 0 0 0 0 0 0 0 0

415.1183 -27.2030 -15.9983 C 0 0 0 0 0 0 0 0 0 0 0 0

416.5069 -24.3738 -17.0584 C 0 0 0 0 0 0 0 0 0 0 0 0

416.0762 -23.1857 -17.4792 C 0 0 0 0 0 0 0 0 0 0 0 0

414.8603 -22.8724 -17.7279 N 0 0 0 0 0 0 0 0 0 0 0 0

414.8456 -21.6284 -17.9776 C 0 0 0 0 0 0 0 0 0 0 0 0

416.1589 -20.9126 -17.7699 C 0 0 0 0 0 0 0 0 0 0 0 0

417.1033 -22.0995 -17.7037 C 0 0 0 0 0 0 0 0 0 0 0 0

413.8066 -20.8880 -18.3558 C 0 0 0 0 0 0 0 0 0 0 0 0

409.2415 -21.1002 -19.0339 C 0 0 0 0 0 0 0 0 0 0 0 0

409.0263 -23.4546 -18.1314 H 0 0 0 0 0 0 0 0 0 0 0 0

410.0162 -27.8550 -15.7795 C 0 0 0 0 0 0 0 0 0 0 0 0

412.6131 -27.9242 -15.8489 H 0 0 0 0 0 0 0 0 0 0 0 0

417.4686 -26.3495 -16.1774 C 0 0 0 0 0 0 0 0 0 0 0 0

415.1888 -28.5892 -15.4328 C 0 0 0 0 0 0 0 0 0 0 0 0

417.8653 -24.9532 -16.7162 C 0 0 0 0 0 0 0 0 0 0 0 0

416.3838 -20.2424 -18.6318 H 0 0 0 0 0 0 0 0 0 0 0 0

417.8059 -22.0316 -16.8394 H 0 0 0 0 0 0 0 0 0 0 0 0

413.9916 -19.8271 -18.5917 H 0 0 0 0 0 0 0 0 0 0 0 0

411.5433 -20.6081 -18.8447 C 0 0 0 0 0 0 0 0 0 0 0 0

409.9287 -29.0000 -16.7952 C 0 0 0 0 0 0 0 0 0 0 0 0

416.1455 -20.0926 -16.4716 C 0 0 0 0 0 0 0 0 0 0 0 0

409.0193 -27.6929 -15.3095 H 0 0 0 0 0 0 0 0 0 0 0 0

410.6715 -28.1378 -14.9238 H 0 0 0 0 0 0 0 0 0 0 0 0

418.2161 -27.2092 -15.7777 O 0 0 0 0 0 0 0 0 0 0 0 0

416.2403 -28.9114 -15.2626 H 0 0 0 0 0 0 0 0 0 0 0 0

414.7229 -29.3195 -16.1325 H 0 0 0 0 0 0 0 0 0 0 0 0

414.6577 -28.6364 -14.4551 H 0 0 0 0 0 0 0 0 0 0 0 0

418.3797 -24.3618 -15.9239 H 0 0 0 0 0 0 0 0 0 0 0 0

418.5115 -25.0748 -17.6149 H 0 0 0 0 0 0 0 0 0 0 0 0

410.9221 -29.2144 -17.2519 H 0 0 0 0 0 0 0 0 0 0 0 0

409.2206 -28.7524 -17.6195 H 0 0 0 0 0 0 0 0 0 0 0 0

409.5708 -29.9379 -16.3108 H 0 0 0 0 0 0 0 0 0 0 0 0

411.5632 -19.1898 -19.3406 C 0 0 0 0 0 0 0 0 0 0 0 0

411.8741 -18.4974 -18.5256 H 0 0 0 0 0 0 0 0 0 0 0 0

412.2679 -19.0832 -20.1965 H 0 0 0 0 0 0 0 0 0 0 0 0

417.8845 -22.3202 -19.0154 C 0 0 0 0 0 0 0 0 0 0 0 0

417.2200 -22.1541 -19.8961 H 0 0 0 0 0 0 0 0 0 0 0 0

418.2018 -23.3863 -19.0822 H 0 0 0 0 0 0 0 0 0 0 0 0

415.8998 -20.7292 -15.5907 H 0 0 0 0 0 0 0 0 0 0 0 0

415.3950 -19.2703 -16.5156 H 0 0 0 0 0 0 0 0 0 0 0 0

417.1400 -19.6249 -16.2891 H 0 0 0 0 0 0 0 0 0 0 0 0

410.5617 -18.8587 -19.6971 H 0 0 0 0 0 0 0 0 0 0 0 0

408.2099 -25.6520 -17.0854 C 0 0 0 0 0 0 0 0 0 0 0 0

417.5129 -18.1810 -21.4792 H 0 0 0 0 0 0 0 0 0 0 0 0

407.8843 -25.4900 -18.1381 H 0 0 0 0 0 0 0 0 0 0 0 0

407.7571 -26.6185 -16.7739 H 0 0 0 0 0 0 0 0 0 0 0 0

407.7743 -24.8537 -16.4424 H 0 0 0 0 0 0 0 0 0 0 0 0

408.0835 -20.8689 -19.3087 C 0 0 0 0 0 0 0 0 0 0 0 0

406.8186 -20.6853 -19.6215 C 0 0 0 0 0 0 0 0 0 0 0 0

406.4805 -19.8884 -20.6477 C 0 0 0 0 0 0 0 0 0 0 0 0

405.1905 -19.7402 -21.0005 C 0 0 0 0 0 0 0 0 0 0 0 0

404.2317 -20.3933 -20.3151 C 0 0 0 0 0 0 0 0 0 0 0 0

404.5738 -21.1650 -19.2680 C 0 0 0 0 0 0 0 0 0 0 0 0

405.8617 -21.3175 -18.9253 C 0 0 0 0 0 0 0 0 0 0 0 0

407.2764 -19.3773 -21.2180 H 0 0 0 0 0 0 0 0 0 0 0 0

404.8399 -18.9701 -22.0453 C 0 0 0 0 0 0 0 0 0 0 0 0

403.8070 -21.7144 -18.6938 H 0 0 0 0 0 0 0 0 0 0 0 0

406.1252 -21.9823 -18.0832 H 0 0 0 0 0 0 0 0 0 0 0 0

413.3296 -24.1278 -18.0830 Zn 0 0 0 0 0 0 0 0 0 0 0 0

402.9490 -20.2902 -20.7055 C 0 0 0 0 0 0 0 0 0 0 0 0

403.5582 -18.8863 -22.4302 C 0 0 0 0 0 0 0 0 0 0 0 0

402.5980 -19.5621 -21.7795 C 0 0 0 0 0 0 0 0 0 0 0 0

405.6012 -18.4182 -22.6244 H 0 0 0 0 0 0 0 0 0 0 0 0

402.1613 -20.8378 -20.1602 H 0 0 0 0 0 0 0 0 0 0 0 0

403.2999 -18.2698 -23.3046 H 0 0 0 0 0 0 0 0 0 0 0 0

401.1614 -19.5218 -22.2415 C 0 0 0 0 0 0 0 0 0 0 0 0

405.1625 -20.4250 -28.5067 C 0 0 0 0 0 0 0 0 0 0 0 0

404.3814 -19.4102 -29.3153 C 0 0 0 0 0 0 0 0 0 0 0 0

403.5025 -20.0426 -30.1110 O 0 0 0 0 0 0 0 0 0 0 0 0

404.5311 -18.2122 -29.2569 O 0 0 0 0 0 0 0 0 0 0 0 0

405.6009 -21.1669 -29.2160 H 0 0 0 0 0 0 0 0 0 0 0 0

406.0227 -19.9052 -28.0227 H 0 0 0 0 0 0 0 0 0 0 0 0

402.6768 -19.2355 -30.9136 C 0 0 0 0 0 0 0 0 0 0 0 0

403.3049 -18.6467 -31.6216 H 0 0 0 0 0 0 0 0 0 0 0 0

402.0512 -18.5790 -30.2652 H 0 0 0 0 0 0 0 0 0 0 0 0

399.2222 -20.8409 -25.4308 C 0 0 0 0 0 0 0 0 0 0 0 0

399.3536 -21.6214 -24.4349 N 0 0 0 0 0 0 0 0 0 0 0 0

398.2246 -22.0146 -24.0021 C 0 0 0 0 0 0 0 0 0 0 0 0

397.2903 -21.4303 -24.7669 C 0 0 0 0 0 0 0 0 0 0 0 0

397.9785 -22.8514 -22.9911 C 0 0 0 0 0 0 0 0 0 0 0 0

398.9193 -23.4334 -22.2371 C 0 0 0 0 0 0 0 0 0 0 0 0

400.1753 -23.2569 -22.3206 N 0 0 0 0 0 0 0 0 0 0 0 0

400.7399 -23.9694 -21.4275 C 0 0 0 0 0 0 0 0 0 0 0 0

399.8135 -24.6574 -20.7357 C 0 0 0 0 0 0 0 0 0 0 0 0

398.6330 -24.3146 -21.2626 C 0 0 0 0 0 0 0 0 0 0 0 0

402.0443 -24.0940 -21.1384 C 0 0 0 0 0 0 0 0 0 0 0 0

403.0110 -23.4839 -21.8238 C 0 0 0 0 0 0 0 0 0 0 0 0

402.6899 -22.6940 -22.8695 N 0 0 0 0 0 0 0 0 0 0 0 0

404.8704 -22.8251 -22.6002 C 0 0 0 0 0 0 0 0 0 0 0 0

404.3508 -23.5799 -21.6296 C 0 0 0 0 0 0 0 0 0 0 0 0

404.2568 -21.5618 -24.3174 C 0 0 0 0 0 0 0 0 0 0 0 0

403.3391 -20.9993 -25.1127 C 0 0 0 0 0 0 0 0 0 0 0 0

402.0691 -21.0649 -24.9788 N 0 0 0 0 0 0 0 0 0 0 0 0

401.5055 -20.3583 -25.8687 C 0 0 0 0 0 0 0 0 0 0 0 0

402.4709 -19.5636 -26.7153 C 0 0 0 0 0 0 0 0 0 0 0 0

403.7925 -20.2190 -26.3269 C 0 0 0 0 0 0 0 0 0 0 0 0

400.1965 -20.2363 -26.1115 C 0 0 0 0 0 0 0 0 0 0 0 0

395.9862 -21.5162 -24.6468 C 0 0 0 0 0 0 0 0 0 0 0 0

396.9195 -23.0729 -22.7922 H 0 0 0 0 0 0 0 0 0 0 0 0

400.0148 -25.6256 -19.5953 C 0 0 0 0 0 0 0 0 0 0 0 0

402.3709 -24.7495 -20.3168 H 0 0 0 0 0 0 0 0 0 0 0 0

406.0837 -22.4460 -23.0541 C 0 0 0 0 0 0 0 0 0 0 0 0

405.0858 -24.3583 -20.5803 C 0 0 0 0 0 0 0 0 0 0 0 0

405.7768 -21.5516 -24.2858 C 0 0 0 0 0 0 0 0 0 0 0 0

402.2366 -19.7136 -27.7953 H 0 0 0 0 0 0 0 0 0 0 0 0

404.5626 -19.4676 -26.0302 H 0 0 0 0 0 0 0 0 0 0 0 0

399.9147 -19.5753 -26.9461 H 0 0 0 0 0 0 0 0 0 0 0 0

397.9128 -20.7050 -25.7023 C 0 0 0 0 0 0 0 0 0 0 0 0

400.4131 -27.0154 -20.1075 C 0 0 0 0 0 0 0 0 0 0 0 0

402.4100 -18.0641 -26.3945 C 0 0 0 0 0 0 0 0 0 0 0 0

399.0799 -25.7132 -18.9953 H 0 0 0 0 0 0 0 0 0 0 0 0

400.7735 -25.2351 -18.8784 H 0 0 0 0 0 0 0 0 0 0 0 0

407.1743 -22.7313 -22.6219 O 0 0 0 0 0 0 0 0 0 0 0 0

406.1617 -24.0759 -20.5380 H 0 0 0 0 0 0 0 0 0 0 0 0

405.0220 -25.4480 -20.7992 H 0 0 0 0 0 0 0 0 0 0 0 0

404.6556 -24.1735 -19.5705 H 0 0 0 0 0 0 0 0 0 0 0 0

406.1752 -20.5236 -24.1256 H 0 0 0 0 0 0 0 0 0 0 0 0

406.2315 -22.0139 -25.1903 H 0 0 0 0 0 0 0 0 0 0 0 0

401.3668 -26.9850 -20.6824 H 0 0 0 0 0 0 0 0 0 0 0 0

399.6268 -27.4349 -20.7766 H 0 0 0 0 0 0 0 0 0 0 0 0

400.5538 -27.7274 -19.2616 H 0 0 0 0 0 0 0 0 0 0 0 0

397.2557 -19.9270 -26.8093 C 0 0 0 0 0 0 0 0 0 0 0 0

397.7544 -18.9527 -27.0009 H 0 0 0 0 0 0 0 0 0 0 0 0

397.2887 -20.5214 -27.7502 H 0 0 0 0 0 0 0 0 0 0 0 0

404.3358 -21.1498 -27.4310 C 0 0 0 0 0 0 0 0 0 0 0 0

403.5043 -21.7298 -27.8964 H 0 0 0 0 0 0 0 0 0 0 0 0

405.0040 -21.9173 -26.9796 H 0 0 0 0 0 0 0 0 0 0 0 0

402.5799 -17.8712 -25.3129 H 0 0 0 0 0 0 0 0 0 0 0 0

401.4202 -17.6260 -26.6586 H 0 0 0 0 0 0 0 0 0 0 0 0

403.1838 -17.5023 -26.9652 H 0 0 0 0 0 0 0 0 0 0 0 0

396.1887 -19.7026 -26.5845 H 0 0 0 0 0 0 0 0 0 0 0 0

397.3063 -24.8656 -20.8126 C 0 0 0 0 0 0 0 0 0 0 0 0

402.0162 -19.9220 -31.4890 H 0 0 0 0 0 0 0 0 0 0 0 0

397.3386 -25.9788 -20.8095 H 0 0 0 0 0 0 0 0 0 0 0 0

397.0830 -24.5217 -19.7783 H 0 0 0 0 0 0 0 0 0 0 0 0

396.4434 -24.5867 -21.4538 H 0 0 0 0 0 0 0 0 0 0 0 0

394.7754 -21.5043 -24.5881 C 0 0 0 0 0 0 0 0 0 0 0 0

393.4623 -21.4288 -24.6072 C 0 0 0 0 0 0 0 0 0 0 0 0

392.8724 -20.3550 -25.1573 C 0 0 0 0 0 0 0 0 0 0 0 0

391.5322 -20.2702 -25.2278 C 0 0 0 0 0 0 0 0 0 0 0 0

390.7762 -21.2695 -24.7326 C 0 0 0 0 0 0 0 0 0 0 0 0

391.3748 -22.3350 -24.1699 C 0 0 0 0 0 0 0 0 0 0 0 0

392.7121 -22.4201 -24.1043 C 0 0 0 0 0 0 0 0 0 0 0 0

393.5022 -19.5455 -25.5686 H 0 0 0 0 0 0 0 0 0 0 0 0

390.7770 -23.1693 -23.7664 H 0 0 0 0 0 0 0 0 0 0 0 0

393.1837 -23.3103 -23.6513 H 0 0 0 0 0 0 0 0 0 0 0 0

401.0402 -21.9774 -23.5426 Zn 0 0 0 0 0 0 0 0 0 0 0 0

401.0232 -19.1592 -23.2855 H 0 0 0 0 0 0 0 0 0 0 0 0

400.5984 -18.8381 -21.5663 H 0 0 0 0 0 0 0 0 0 0 0 0

390.9327 -19.2104 -25.7985 C 0 0 0 0 0 0 0 0 0 0 0 0

389.4360 -21.1886 -24.8141 C 0 0 0 0 0 0 0 0 0 0 0 0

389.5956 -19.1451 -25.8779 C 0 0 0 0 0 0 0 0 0 0 0 0

388.8306 -20.1329 -25.3850 C 0 0 0 0 0 0 0 0 0 0 0 0

387.3280 -20.0390 -25.4677 C 0 0 0 0 0 0 0 0 0 0 0 0

386.9051 -20.1442 -26.8000 O 0 0 0 0 0 0 0 0 0 0 0 0

391.5270 -18.3771 -26.2128 H 0 0 0 0 0 0 0 0 0 0 0 0

388.8096 -22.0007 -24.4075 H 0 0 0 0 0 0 0 0 0 0 0 0

389.1207 -18.2675 -26.3491 H 0 0 0 0 0 0 0 0 0 0 0 0

386.8083 -20.8508 -24.9105 H 0 0 0 0 0 0 0 0 0 0 0 0

386.9774 -19.0568 -25.0757 H 0 0 0 0 0 0 0 0 0 0 0 0

403.8822 -22.3211 -23.3094 C 0 0 0 0 0 0 0 0 0 0 0 0

387.1373 -19.3430 -27.2364 H 0 0 0 0 0 0 0 0 0 0 0 0

399.6206 -20.6918 -22.1973 H 0 0 0 0 0 0 0 0 0 0 0 0

1 2 1 0 0 0 0

1 5 1 0 0 0 0

1 6 1 0 0 0 0

1 60 1 0 0 0 0

2 3 1 0 0 0 0

2 4 2 0 0 0 0

3 7 1 0 0 0 0

7 8 1 0 0 0 0

7 9 1 0 0 0 0

7 68 1 0 0 0 0

10 11 1 0 0 0 0

10 32 1 0 0 0 0

10 43 2 0 0 0 0

11 12 1 0 0 0 0

11 82 1 0 0 0 0

12 13 2 0 0 0 0

12 14 1 0 0 0 0

13 33 1 0 0 0 0

13 43 1 0 0 0 0

14 15 2 0 0 0 0

14 34 1 0 0 0 0

15 16 1 0 0 0 0

15 19 1 0 0 0 0

16 17 2 0 0 0 0

16 82 1 0 0 0 0

17 18 1 0 0 0 0

17 20 1 0 0 0 0

18 19 2 0 0 0 0

18 35 1 0 0 0 0

19 67 1 0 0 0 0

20 21 2 0 0 0 0

20 36 1 0 0 0 0

21 22 1 0 0 0 0

21 25 1 0 0 0 0

22 23 1 0 0 0 0

22 82 1 0 0 0 0

23 24 1 0 0 0 0

23 26 2 0 0 0 0

24 25 2 0 0 0 0

24 37 1 0 0 0 0

25 38 1 0 0 0 0

26 27 1 0 0 0 0

26 39 1 0 0 0 0

27 28 2 0 0 0 0

27 31 1 0 0 0 0

28 29 1 0 0 0 0

28 82 1 0 0 0 0

29 30 1 0 0 0 0

29 32 2 0 0 0 0

30 31 1 0 0 0 0

30 40 1 0 0 0 0

30 45 1 0 0 0 0

31 41 1 0 0 0 0

31 60 1 0 0 0 0

32 42 1 0 0 0 0

33 72 3 0 0 0 0

35 44 1 0 0 0 0

35 46 1 0 0 0 0

35 47 1 0 0 0 0

37 39 1 0 0 0 0

37 48 2 0 0 0 0

38 49 1 0 0 0 0

38 50 1 0 0 0 0

38 51 1 0 0 0 0

39 52 1 0 0 0 0

39 53 1 0 0 0 0

43 57 1 0 0 0 0

44 54 1 0 0 0 0

44 55 1 0 0 0 0

44 56 1 0 0 0 0

45 63 1 0 0 0 0

45 64 1 0 0 0 0

45 65 1 0 0 0 0

57 58 1 0 0 0 0

57 59 1 0 0 0 0

57 66 1 0 0 0 0

60 61 1 0 0 0 0

60 62 1 0 0 0 0

67 69 1 0 0 0 0

67 70 1 0 0 0 0

67 71 1 0 0 0 0

72 73 1 0 0 0 0

73 74 2 0 0 0 0

73 78 1 0 0 0 0

74 75 1 0 0 0 0

74 79 1 0 0 0 0

75 76 2 0 0 0 0

75 83 1 0 0 0 0

76 77 1 0 0 0 0

76 84 1 0 0 0 0

77 78 2 0 0 0 0

77 80 1 0 0 0 0

78 81 1 0 0 0 0

82 94 1 0 0 0 0

83 85 2 0 0 0 0

83 89 1 0 0 0 0

84 86 2 0 0 0 0

84 90 1 0 0 0 0

85 86 1 0 0 0 0

85 91 1 0 0 0 0

86 87 1 0 0 0 0

87 88 1 0 0 0 0

87 92 1 0 0 0 0

87 93 1 0 0 0 0

88188 1 0 0 0 0

94184 1 0 0 0 0

94187 1 0 0 0 0

95 96 1 0 0 0 0

95 99 1 0 0 0 0

95100 1 0 0 0 0

95154 1 0 0 0 0

96 97 1 0 0 0 0

96 98 2 0 0 0 0

97101 1 0 0 0 0

101102 1 0 0 0 0

101103 1 0 0 0 0

101162 1 0 0 0 0

104105 1 0 0 0 0

104126 1 0 0 0 0

104137 2 0 0 0 0

105106 1 0 0 0 0

105177 1 0 0 0 0

106107 2 0 0 0 0

106108 1 0 0 0 0

107127 1 0 0 0 0

107137 1 0 0 0 0

108109 2 0 0 0 0

108128 1 0 0 0 0

109110 1 0 0 0 0

109113 1 0 0 0 0

110111 2 0 0 0 0

110177 1 0 0 0 0

111112 1 0 0 0 0

111114 1 0 0 0 0

112113 2 0 0 0 0

112129 1 0 0 0 0

113161 1 0 0 0 0

114115 2 0 0 0 0

114130 1 0 0 0 0

115116 1 0 0 0 0

115119 1 0 0 0 0

116117 1 0 0 0 0

116177 1 0 0 0 0

117118 1 0 0 0 0

117120 2 0 0 0 0

118119 2 0 0 0 0

118131 1 0 0 0 0

119132 1 0 0 0 0

120121 1 0 0 0 0

120133 1 0 0 0 0

121122 2 0 0 0 0

121125 1 0 0 0 0

122123 1 0 0 0 0

122177 1 0 0 0 0

123124 1 0 0 0 0

123126 2 0 0 0 0

124125 1 0 0 0 0

124134 1 0 0 0 0

124139 1 0 0 0 0

125135 1 0 0 0 0

125154 1 0 0 0 0

126136 1 0 0 0 0

127166 3 0 0 0 0

129138 1 0 0 0 0

129140 1 0 0 0 0

129141 1 0 0 0 0

131133 1 0 0 0 0

131142 2 0 0 0 0

132143 1 0 0 0 0

132144 1 0 0 0 0

132145 1 0 0 0 0

133146 1 0 0 0 0

133147 1 0 0 0 0

137151 1 0 0 0 0

138148 1 0 0 0 0

138149 1 0 0 0 0

138150 1 0 0 0 0

139157 1 0 0 0 0

139158 1 0 0 0 0

139159 1 0 0 0 0

151152 1 0 0 0 0

151153 1 0 0 0 0

151160 1 0 0 0 0

154155 1 0 0 0 0

154156 1 0 0 0 0

161163 1 0 0 0 0

161164 1 0 0 0 0

161165 1 0 0 0 0

166167 1 0 0 0 0

167168 2 0 0 0 0

167172 1 0 0 0 0

168169 1 0 0 0 0

168173 1 0 0 0 0

169170 2 0 0 0 0

169174 1 0 0 0 0

170171 1 0 0 0 0

170178 1 0 0 0 0

171172 2 0 0 0 0

171175 1 0 0 0 0

172176 1 0 0 0 0

174179 2 0 0 0 0

174181 1 0 0 0 0

178180 2 0 0 0 0

178182 1 0 0 0 0

179180 1 0 0 0 0

179183 1 0 0 0 0

180184 1 0 0 0 0

184185 1 0 0 0 0

184186 1 0 0 0 0

189279 1 0 0 0 0

189360 1 0 0 0 0

189376 1 0 0 0 0

190191 1 0 0 0 0

190194 1 0 0 0 0

190195 1 0 0 0 0

190249 1 0 0 0 0

191192 1 0 0 0 0

191193 2 0 0 0 0

192196 1 0 0 0 0

196197 1 0 0 0 0

196198 1 0 0 0 0

196257 1 0 0 0 0

199200 1 0 0 0 0

199221 1 0 0 0 0

199232 2 0 0 0 0

200201 1 0 0 0 0

200272 1 0 0 0 0

201202 2 0 0 0 0

201203 1 0 0 0 0

202222 1 0 0 0 0

202232 1 0 0 0 0

203204 2 0 0 0 0

203223 1 0 0 0 0

204205 1 0 0 0 0

204208 1 0 0 0 0

205206 2 0 0 0 0

205272 1 0 0 0 0

206207 1 0 0 0 0

206209 1 0 0 0 0

207208 2 0 0 0 0

207224 1 0 0 0 0

208256 1 0 0 0 0

209210 2 0 0 0 0

209225 1 0 0 0 0

210211 1 0 0 0 0

210214 1 0 0 0 0

211212 1 0 0 0 0

211272 1 0 0 0 0

212213 1 0 0 0 0

212215 2 0 0 0 0

213214 2 0 0 0 0

213226 1 0 0 0 0

214227 1 0 0 0 0

215216 1 0 0 0 0

215228 1 0 0 0 0

216217 2 0 0 0 0

216220 1 0 0 0 0

217218 1 0 0 0 0

217272 1 0 0 0 0

218219 1 0 0 0 0

218221 2 0 0 0 0

219220 1 0 0 0 0

219229 1 0 0 0 0

219234 1 0 0 0 0

220230 1 0 0 0 0

220249 1 0 0 0 0

221231 1 0 0 0 0

222261 3 0 0 0 0

224233 1 0 0 0 0

224235 1 0 0 0 0

224236 1 0 0 0 0

226228 1 0 0 0 0

226237 2 0 0 0 0

227238 1 0 0 0 0

227239 1 0 0 0 0

227240 1 0 0 0 0

228241 1 0 0 0 0

228242 1 0 0 0 0

232246 1 0 0 0 0

233243 1 0 0 0 0

233244 1 0 0 0 0

233245 1 0 0 0 0

234252 1 0 0 0 0

234253 1 0 0 0 0

234254 1 0 0 0 0

246247 1 0 0 0 0

246248 1 0 0 0 0

246255 1 0 0 0 0

249250 1 0 0 0 0

249251 1 0 0 0 0

256258 1 0 0 0 0

256259 1 0 0 0 0

256260 1 0 0 0 0

261262 1 0 0 0 0

262263 2 0 0 0 0

262267 1 0 0 0 0

263264 1 0 0 0 0

263268 1 0 0 0 0

264265 2 0 0 0 0

264269 1 0 0 0 0

265266 1 0 0 0 0

265273 1 0 0 0 0

266267 2 0 0 0 0

266270 1 0 0 0 0

267271 1 0 0 0 0

269274 2 0 0 0 0

269276 1 0 0 0 0

273275 2 0 0 0 0

273277 1 0 0 0 0

274275 1 0 0 0 0

274278 1 0 0 0 0

275279 1 0 0 0 0

279361 1 0 0 0 0

279362 1 0 0 0 0

280281 1 0 0 0 0

280284 1 0 0 0 0

280285 1 0 0 0 0

280338 1 0 0 0 0

281282 1 0 0 0 0

281283 2 0 0 0 0

282286 1 0 0 0 0

286287 1 0 0 0 0

286288 1 0 0 0 0

286346 1 0 0 0 0

289290 1 0 0 0 0

289310 1 0 0 0 0

289321 2 0 0 0 0

290291 1 0 0 0 0

290360 1 0 0 0 0

291292 2 0 0 0 0

291293 1 0 0 0 0

292311 1 0 0 0 0

292321 1 0 0 0 0

293294 2 0 0 0 0

293312 1 0 0 0 0

294295 1 0 0 0 0

294298 1 0 0 0 0

295296 2 0 0 0 0

295360 1 0 0 0 0

296297 1 0 0 0 0

296299 1 0 0 0 0

297298 2 0 0 0 0

297313 1 0 0 0 0

298345 1 0 0 0 0

299300 2 0 0 0 0

299314 1 0 0 0 0

300301 1 0 0 0 0

300303 1 0 0 0 0

301360 1 0 0 0 0

301374 1 0 0 0 0

302303 2 0 0 0 0

302315 1 0 0 0 0

302374 1 0 0 0 0

303316 1 0 0 0 0

304305 1 0 0 0 0

304317 1 0 0 0 0

304374 2 0 0 0 0

305306 2 0 0 0 0

305309 1 0 0 0 0

306307 1 0 0 0 0

306360 1 0 0 0 0

307308 1 0 0 0 0

307310 2 0 0 0 0

308309 1 0 0 0 0

308318 1 0 0 0 0

308323 1 0 0 0 0

309319 1 0 0 0 0

309338 1 0 0 0 0

310320 1 0 0 0 0

311350 3 0 0 0 0

313322 1 0 0 0 0

313324 1 0 0 0 0

313325 1 0 0 0 0

315317 1 0 0 0 0

315326 2 0 0 0 0

316327 1 0 0 0 0

316328 1 0 0 0 0

316329 1 0 0 0 0

317330 1 0 0 0 0

317331 1 0 0 0 0

321335 1 0 0 0 0

322332 1 0 0 0 0

322333 1 0 0 0 0

322334 1 0 0 0 0

323341 1 0 0 0 0

323342 1 0 0 0 0

323343 1 0 0 0 0

335336 1 0 0 0 0

335337 1 0 0 0 0

335344 1 0 0 0 0

338339 1 0 0 0 0

338340 1 0 0 0 0

345347 1 0 0 0 0

345348 1 0 0 0 0

345349 1 0 0 0 0

350351 1 0 0 0 0

351352 2 0 0 0 0

351356 1 0 0 0 0

352353 1 0 0 0 0

352357 1 0 0 0 0

353354 2 0 0 0 0

353363 1 0 0 0 0

354355 1 0 0 0 0

354364 1 0 0 0 0

355356 2 0 0 0 0

355358 1 0 0 0 0

356359 1 0 0 0 0

363365 2 0 0 0 0

363369 1 0 0 0 0

364366 2 0 0 0 0

364370 1 0 0 0 0

365366 1 0 0 0 0

365371 1 0 0 0 0

366367 1 0 0 0 0

367368 1 0 0 0 0

367372 1 0 0 0 0

367373 1 0 0 0 0

368375 1 0 0 0 0

M END
